# Genome editing with removable TALEN vectors harboring a yeast centromere and autonomous replication sequence in oleaginous microalga

**DOI:** 10.1038/s41598-022-06495-y

**Published:** 2022-02-15

**Authors:** Tomokazu Kurita, Masako Iwai, Keishi Moroi, Kumiko Okazaki, Seiji Nomura, Fumihiko Saito, Shinichiro Maeda, Akihide Takami, Atsushi Sakamoto, Hiroyuki Ohta, Tetsushi Sakuma, Takashi Yamamoto

**Affiliations:** 1 Division of Integrated Sciences for Life, Graduate School of Integrated Sciences for Life, Hiroshima University, 1-3-1 Kagamiyama, Higashi-Hiroshima, Hiroshima 739-8526 Japan; 2 School of Life Science and Technology, Tokyo Institute of Technology, 4259-B-65 Nagatsuta-cho, Midori-ku, Yokohama, Kanagawa 226-8501 Japan; 3 Mazda Motor Corporation, 3-1 Shinchi, Fuchu-cho, Aki-gun, Hiroshima, 730-8670 Japan

**Keywords:** Industrial microbiology, Molecular engineering

## Abstract

Algal lipids are expected to become a basis for sustainable fuels because of the highly efficient lipid production by photosynthesis accompanied by carbon dioxide assimilation. Molecular breeding of microalgae has been studied to improve algal lipid production, but the resultant gene-modified algae containing transgenes are rarely used for outdoor culture because the use of genetically modified organisms (GMOs) is strictly restricted under biocontainment regulations. Recently, it was reported that plasmids containing yeast centromere and autonomous replication sequence (CEN/ARS) behaved as episomes in *Nannochloropsis* species. We previously reported that the Platinum TALEN (PtTALEN) system exhibited high activity in *Nannochloropsis oceanica*. Therefore, we attempted to develop a genome editing system in which the expression vectors for PtTALEN can be removed from host cells after introduction of mutations. Using all-in-one PtTALEN plasmids containing CEN/ARS, targeted mutations and removal of all-in-one vectors were observed in *N. oceanica*, suggesting that our all-in-one PtTALEN vectors enable the construction of mutated *N. oceanica* without any transgenes. This system will be a feasible method for constructing non-GMO high-performance algae.

## Introduction

The development of sustainable energy production and reduction of carbon dioxide emissions are vital to protect the environment. Biodiesels are expected to become a new sustainable source of energy. Biodiesels are made from triacylglycerols accumulated in plant seeds or algal cells, which are produced by photosynthesis accompanying carbon dioxide assimilation. Microalgae are especially promising as producers of biodiesel feedstocks due to their higher productivity than terrestrial plants without impeding food production^1,2^. Despite these inherent advantages, the practical application of algal biodiesels has not yet been achieved, in part because biodiesel production costs are higher than for fossil fuels^3^. Thus, there is a need to improve the lipid productivity of microalgae using molecular breeding for their practical use. Many researchers have attempted to generate algae that accumulate excess lipids using molecular breeding of various microalgal species to date.

*Nannochloropsis oceanica* is a secondary symbiosis heterokont eukaryotic marine alga. *Nannochloropsis* species accumulate the highest amounts of lipids (> 60% of dry weight) among microalgae^1^. *Botryococcus* also accumulates as much lipids as *Nannochloropsis* species (75% of dry weight)^1^, but gene manipulation systems for *Botryococcus* are fewer than for *Nannochloropsis* species^3–5^. Most gene manipulation systems for molecular breeding have been developed in model green alga and diatoms such as *Chlamydomonas reinhardtii* and *Phaeodactylum tricornutum*^3,5^. However, the lipid storage levels of these algae (20–30% dry weight) are not comparable to those of *Nannochloropsis* species^1,6^. Therefore, *Nannochloropsis* species are expected to be potent lipid producers for biodiesel and have been used for the development of algae with high lipid production. Ajjwai et al*.* reported that strains in which RNAi attenuated levels of the potential transcription factor gene *ZnCys* accumulated twice as much lipid as the wild-type strains of *Nannochloropsis* species^7^.

Recently, genome editing systems have been used for gene manipulation in various species because of their high efficiency and ease of use. In particular, the clustered regularly interspaced short palindromic repeats (CRISPR)-CRISPR-associated protein 9 (Cas9) system is being actively used because of its convenience for plasmid construction. The CRISPR-Cas9 system has also been applied to *Nannochloropsis* species^7–12^. In addition to molecular breeding of *Nannochloropsis* species, outdoor cultivation of oleaginous microalgae is vital for obtaining large numbers of algal cells at a low cost. However, in most countries, the outdoor culture of genetically modified organisms (GMOs) is restricted under biocontainment regulations. To overcome this hurdle, development of transgene-free gene manipulation systems is needed. Gene manipulation using episomal plasmids is effective because plasmids containing expression cassettes of genome editing tools can be removed from the host cells. Plasmids containing a yeast centromere and autonomous replication sequence (CEN/ARS) behave as an episome that is an extrachromosomal nuclear DNA. CEN/ARS consists of an endogenous centromere sequence, *CEN6,* and an autonomous replication sequence, *ASRH4*, from the budding yeast, *Saccharomyces cerevisiae*, conferring mitotic and meiotic stability to the plasmids in *S. cerevisiae*^13^. Surprisingly, plasmids containing the same CEN/ARS also behaved as episomes in diatoms^14–16^ and *Nannochloropsis* species^9^. Poliner et al*.* reported that CRISPR-Cas9 all-in-one vectors containing a CEN/ARS could be removed from *Nannochloropsis* species host cells by culturing in F2N medium without antibiotics^9^. This strategy is feasible for constructing plasmid-removed genome edited strains. However, the reported mutagenesis efficiency was not very high and target site selection of the CRISPR-Cas9 system is constrained by the protospacer adjacent motif (PAM) sequence. Poliner et al*.* reported the highest mutagenesis activity of 60% with the CRISPR-Cas9 system in *Nannochloropsis* species^9^. On the other hand, we previously reported that the all-in-one Platinum transcription activator-like effector (TALE) nucleases (PtTALENs) system exhibited high genome editing activity, 100%, in *Nannochloropsis oceanica*^17^.

TALENs are also effective genome editing tools. TALENs consist of the TALE domain, isolated from the plant bacterial pathogen *Xanthomonas* spp*.*^18^, and FokI nuclease domain, isolated from the marine bacterium *Flavobacterium okeanokoites*. The TALE domain typically consists of 15 to 20 TALE repeats, and the TALE repeats bind individual nucleotides. Therefore, the TALE repeat arrangement enables the design of the TALEN binding sequence^19^. PtTALENs, which were constructed by incorporating the variations of the 4th and 32nd amino acid residues in the TALE repeats based on the natural diversity of TALE domains, are highly efficient TALENs^20^. On the other hand, all-in-one PtTALEN plasmids may be inserted into the endogenous genome of the host *N. oceanica* cells. This is inconvenient for outdoor culture and serial gene manipulation, since transformant cells have transgenes containing antibiotic marker genes. In addition, the locus effect of plasmid insertion could negatively affect host cell integrity and PtTALEN expression.

In this study, we attempted to construct an all-in-one PtTALEN containing CEN/ARS to confer removability and reduce the risk of plasmid insertion. The resultant all-in-one PtTALEN-ARS vectors exhibited genome editing activity in and removability from *N. oceanica* cells.

## Results

### Construction of all-in-one PtTALEN-ARS plasmids and introduction of mutations in *N. oceanica*

**Figure 1:**
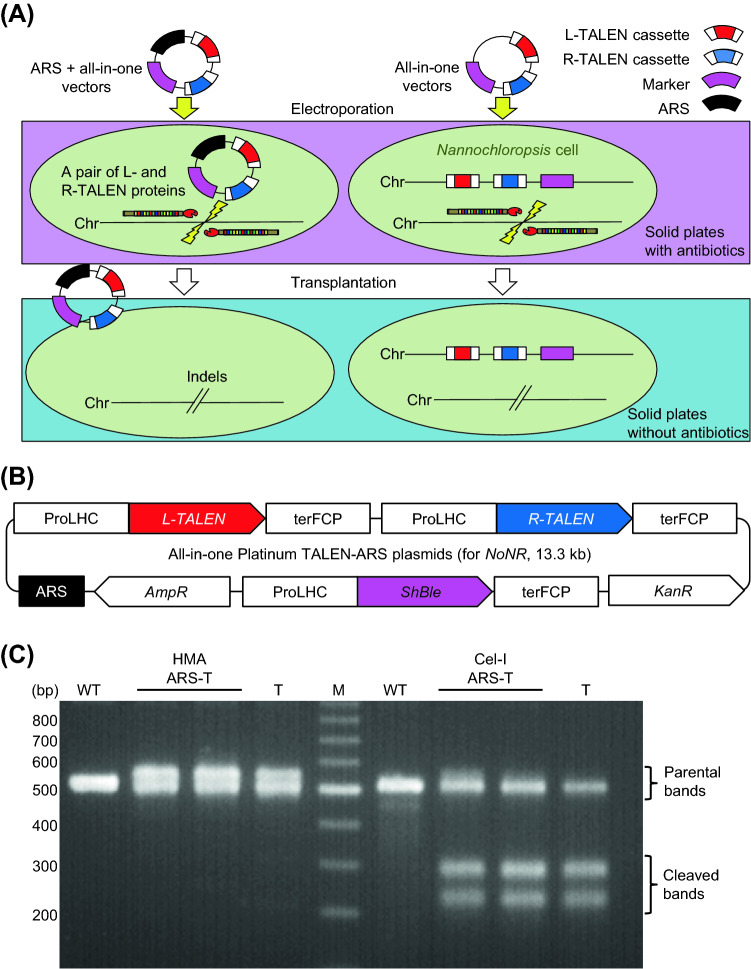
Construction of all-in-one PtTALEN-ARS plasmids and verification of genome editing activity. (**A**) Workflow of transgene-free genome editing using all-in-one PtTALEN-ARS plasmids. The plasmids containing CEN/ARS are not inserted into the chromosome and can be removed after mutagenesis. Chr: chromosome. (**B**) Schematic diagram of all-in-one PtTALEN-ARS plasmids. These plasmids have two antibiotic genes, *AmpR* and *KanR*. These are facilitated to construct the all-in-one TALEN-ARS vectors. ProLHC: LHC promoter; terFCP: FCP terminator; L-TALEN: left TALEN; R-TALEN: right TALEN; *AmpR*: ampicillin resistance gene; *ShBle*: zeocin resistance gene; *KanR*: kanamycin resistance gene; ARS: yeast centromere and autonomous replication sequence. (**C**) Heteroduplex mobility assay (HMA) and Cel-I assay for TALEN target sites of *NoNR* genes in wild-type and TALEN-induced *N. oceanica* by the introduction of all-in-one PtTALEN-ARS or TALEN plasmids targeting *NoNR*. HMA was performed by amplifying the *NoNR* target site. The Cel-I assay was performed by treating *NoNR* PCR products with Cel-I enzymes. WT: Wild-type genomic DNAs; ARS-T: total DNAs from all emerged *N. oceanica* colonies following introduction of all-in-one *NoNR* PtTALEN-ARS plasmids; T: total DNAs from all emerged *N. oceanica* colonies following the introduction of all-in-one *NoNR* PtTALEN plasmids; M: DNA ladder marker.

To construct transgene-free genome edited *N. oceanica*, the vectors used for expressing genome-editing tools must be removed. The all-in-one PtTALEN vectors containing CEN/ARS were expected to behave as episomes in *N. oceanica* cells (Fig. 1A). Under non-selective conditions, the all-in-one PtTALEN vectors containing CEN/ARS may be removed spontaneously from host *N. oceanica* cells, since the vectors are not necessary for host cell growth.

Previously, we reported that the all-in-one PtTALEN plasmids possessed high mutagenesis activity; however, the insertion of the expression cassette of the right-TALEN into the left-TALEN expression plasmids was inefficient. This might be due to the large vector size. To improve the ease of construction of all-in-one PtTALEN plasmids, we initially aimed to use two antibiotic markers. The kanamycin resistance gene was inserted into the flanking region of the right-TALEN expression cassette. The resultant pMD20-TALEN47-KanR plasmid contained only the kanamycin resistance gene as an antibiotic marker. The integrated all-in-one plasmids possess both ampicillin and kanamycin resistance (Supplementary Fig. S1). Therefore, only strains harboring the newly integrated all-in-one plasmids are capable of growing on LB solid plates containing both ampicillin and kanamycin. In practice, colony PCR and restriction enzyme verification showed that all tested colonies harbored the expected all-in-one PtTALEN vectors (Supplementary Fig. S2). Next, we constructed all-in-one PtTALEN-ARS plasmids as shown in Fig. 1B. CEN/ARS was amplified using a yeast low-copy plasmid as a template and then inserted into the left-TALEN expression plasmids. DNA-binding modules for the endogenous nitrate reductase gene *NoNR* were as previously described^17^. Due to concern about the lowering mutagenesis activity by inserting ARS into all-in-one TALEN vectors, we attempted to confirm the genome editing activity of TALENs expressed from all-in-one PtTALEN-ARS plasmids targeting *NoNR*. All-in-one PtTALEN-ARS vectors were introduced into *N. oceanica* cells. All colonies on selective plates were collected, mixed, and subjected to extraction of total DNAs, which potentially contain both genomic and plasmid DNAs. In addition, the region around the *NoNR* target site (516 bp) was amplified using total DNAs. The genome editing activity of the all-in-one PtTALEN-ARS plasmid was assayed using the heteroduplex mobility assay (HMA) and Cel-I assay of the PCR products. The high mutagenesis activity of all-in-one PtTALEN plasmid without ARS was previously reported^17^. Therefore, total DNAs extracted from the colonies treated with all-in-one PtTALEN plasmid lacking ARS was used as a positive control. PCR products derived from all-in-one PtTALEN-ARS plasmid exhibited a band shift in the HMA that was similar to the positive control, suggesting the introduction of mutations (Fig. 1C). Moreover, the Cel-I assay detected cleavage bands for both all-in-one PtTALEN-ARS plasmids and the positive control (Fig. 1C). These results indicated that PtTALENs expressed from all-in-one PtTALEN-ARS plasmids exhibit genome editing activity in *N. oceanica*.

### Clearance of all-in-one PtTALEN-ARS plasmids from *N. oceanica* cells

**Figure 2:**
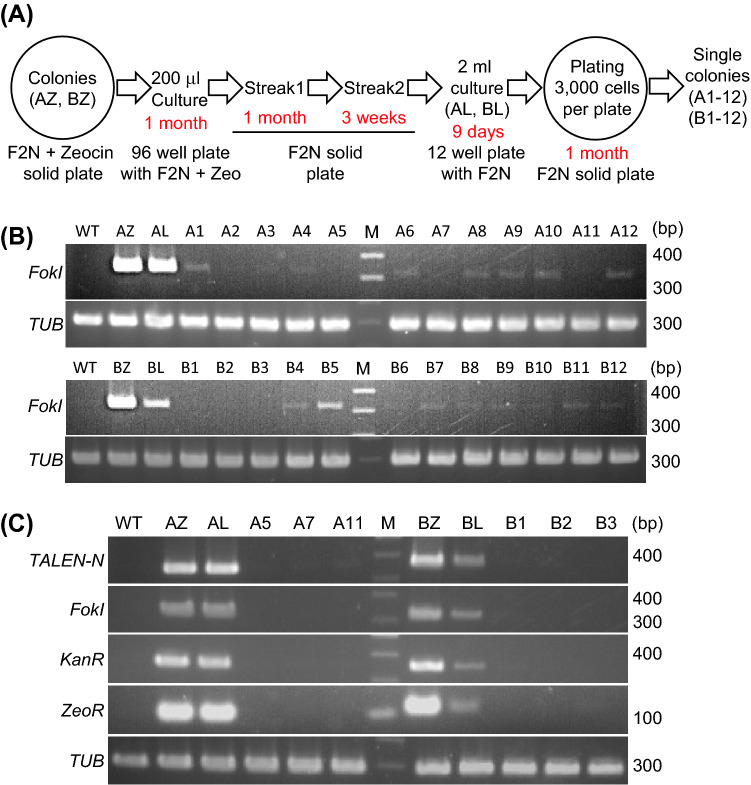
Clearance of all-in-one PtTALEN-ARS plasmids from *Nannochloropsis oceanica* cells. (**A**) Workflow for the clearance of all-in-one PtTALEN-ARS plasmids from host cells. All-in-one PtTALEN-ARS plasmids were removed by using F2N without zeocin solid plate or liquid medium. AZ and BZ are collected single colonies that emerged in a selectable zeocin plate following the introduction of all-in-one *NoNR* PtTALEN-ARS vectors. (**B**) Verification of all-in-one PtTALEN-ARS plasmid clearance by PCR using primers for amplification of *FokI* nuclease of TALENs. All-in-one PtTALEN-ARS plasmids clearance of strains collected from each clearing process was analyzed by using *FokI* primers, the internal plasmid sequence. *FokI*: *FokI* PCR amplicons; *TUB*: *tubulin-beta* PCR amplicons; WT: wild-type genomic DNAs; AZ and BZ: total DNAs from a single colony of *N. oceanica* that emerged in a selectable plate by the introduction of all-in-one *NoNR* PtTALEN-ARS plasmids; AL and BL: total DNA from AZ or BZ treated for plasmid clearance as shown in this figure; A1-A12 and B1-B12: total DNAs from single colonies that emerged in a solid F2N plate by spreading the AL or BL culture solution; M: DNA ladder marker. (**C**) Verification of all-in-one PtTALEN-ARS vector clearance by PCR using four pairs of primers for the detection of the all-in-one PtTALEN-ARS vector. The plasmid clearance of strains that were not detected *FokI* PCR bands was verified again using the other 3 pairs of internal plasmid primers. *PtTALEN-N*: PtTALEN N terminal domain PCR amplicons; *FokI*: *FokI* PCR amplicons; *KanR*: kanamycin resistance gene PCR amplicons; *ZeoR*: zeocin resistance gene (*ShBle*) PCR amplicons; *TUB*: *tubulin-beta* PCR amplicons.

**Figure 3:**
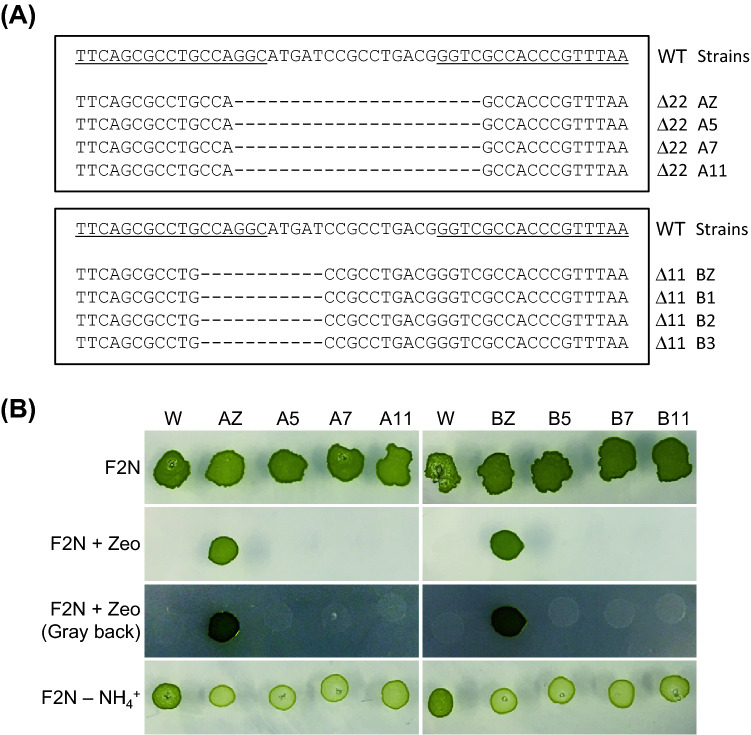
Verification of gene edited *Nannochloropsis oceanica* strains with vector removal. (**A**) *NoNR* target site sequences of strains with potential all-in-one *NoNR* PtTALEN-ARS plasmids removal. To rule out contamination with wild-type cells, the *NoNR* target site sequencing of potentially plasmid removed strains was performed. WT: Wild-type genomic DNAs; AZ and BZ: total DNAs from a single colony of *N. oceanica* that emerged in a selectable plate following introduction of all-in-one *NoNR* PtTALEN-ARS plasmids; A5, A7 and A11: total DNAs of clearance treated cells of AZ; B1, B2 and B3: total DNAs of clearance treated cells of BZ. Underlined sequences of WT strain indicate the TALEN binding sites. Dashes indicate deletions. (**B**) Verification of all-in-one PtTALEN-ARS vector clearance by phenotypic analysis using spot tests. Wild-type, TALEN strains, or potentially plasmid-removed strains were cultured with 2 mL F2N liquid medium, and 5 µL (3.6 × 10^4^ cells µL^−1^) of resultant cultures were spotted onto solid plates. Because the spots of zeocin sensitive strains could not be seen on the F2N + Zeo plate in the picture with a white background, the F2N + Zeo plate pictures with a gray background are presented to show the spot marks. F2N: F2N plate; F2N + Zeo: F2N 50% seawater containing 2 µg L^−1^ zeocin plate; F2N–NH_4_^+^: F2N without ammonium plate.

We attempted to remove the all-in-one PtTALEN-ARS plasmids from transformed cells. Transformants of all-in-one PtTALEN-ARS plasmids targeting *NoNR* were collected from selective plates. The *NoNR* target site of these transformants was confirmed by sequencing. Two *NoNR* mutants (Strain ID: AZ and BZ) were used for the following experiments. To remove the vectors, these strains were cultured using liquid or solid medium in the absence of zeocin, as shown in Fig. 2A. The 12 colonies that appeared on the solid medium without zeocin were collected, and the clearance of all-in-one PtTALEN-ARS plasmids was confirmed by PCR (Fig. 2B). A primer pair for amplification of the *FokI* nuclease domain sequence of TALEN was used. In all tested colonies, the intensity of *FokI* PCR bands was substantially reduced. In particular, six strains derived from AZ or BZ did not have detectable PCR bands for *FokI* (Strain ID: A5, A7, and A11 and Strain ID: B1, B2, and B3). The strains with potential plasmid removal were investigated again with three additional primer pairs for plasmid detection (Fig. 2C). The absence of the all-in-one PtTALEN-ARS plasmid was further verified by the absence of PCR bands for *FokI*, *KanR* and *ZeoR* in these strains. This result suggested that these strains do not harbor all-in-one PtTALEN-ARS plasmids. Subsequently, we checked the sequence of the *NoNR* target site to rule out possible wild-type strain contamination (Fig. 3A). Sequence analysis showed that the *NoNR* target site in these strains (Fig. 3A, Strain ID: A5, A7, A11, B1–B3) corresponded with that of the parental strains (Fig. 3A, Strain ID: AZ, BZ). Furthermore, we performed spot test using each type of solid plate for phenotypic validation (Fig. 3B). *NoNR* mutants were reported to exhibit chlorosis, which is the phenotype of bleached pigments on F2N plates containing nitrate as the sole nitrogen source^8,9,12,17,21^. Chlorosis is a typical phenotype of nitrate reductase mutants caused by depletion of the nitrogen source in *N. oceanica*. We used three plate types: F2N (normal F2N medium containing both nitrate and ammonium as the nitrogen source), F2N 50% seawater containing zeocin (F2N + Zeo), and F2N containing nitrate as the sole nitrogen source (F2N–NH_4_^+^). Because F2N medium has too high salt concentration for zeocin, an F2N 50% seawater medium containing zeocin plate was used as in previous reports^22–24^. The six tested strains with potential plasmid removal exhibited sensitivity to zeocin (F2N + Zeo) as observed for the wild-type, as well as chlorosis on the F2N–NH_4_^+^ plate, which is similar to the parental *NoNR* mutant strains harboring the all-in-one PtTALEN-ARS vectors. This result is consistent with our model (Fig. 1A), as the spot tests showed that the six tested strains exhibited loss of *NoNR* function, which is the TALEN target gene, and zeocin resistance gene, which is the antibiotic marker of the all-in-one PtTALEN-ARS vectors. Accordingly, we successfully obtained plasmid-removed *NoNR* mutant *N. oceanica* cells using all-in-one PtTALEN-ARS vectors.

The effects of all-in-one PtTALEN-ARS vectors on host cell growth and lipid accumulation (Supplementary Fig. S3) were also examined, because they are important for a molecular breeding tool. The *NoNR* frame shift mutants harboring all-in-one PtTALEN-ARS vectors (Strain ID: AZ and BZ) or the mutants removed the vectors (Strain ID: A5 and B1) were used. The lipid contents were assayed by TAG contents per culture and TAG contents per cell because both are important information for algal lipid production. No significant differences in growth among these strains were found at each time point. It was also found that the A5 strain accumulated significantly higher TAG contents than the parental strain, AZ. On the other hand, TAG contents were detected with no significant difference among WT, AZ, BZ, and B1 strains.

To establish the transgene-free genome editing system, electroporation using carrier-DNAs, which are transgenes, was inconvenient because these carrier-DNAs remain in the host cells even after plasmid removal. However, carrier-DNAs are necessary for measurable and efficient electroporation using the Gene Pulser II electroporator. Therefore, an electroporation system was established without carrier-DNAs using the highly efficient Elepo21 electroporator (Supplementary Table S3). We introduced the all-in-one TALEN-ARS vector into *N. oceanica* using the Elepo21. Under 1250–1750 V poring pulse voltage, approximately 1000 colony-forming units per µg DNA were obtained. In accordance with the vector-removable genome editing system and carrier DNA-free electroporation, we successfully established a transgene-free genome editing system.

### Verification of removal efficiency for all-in-one PtTALEN-ARS plasmids

**Figure 4:**
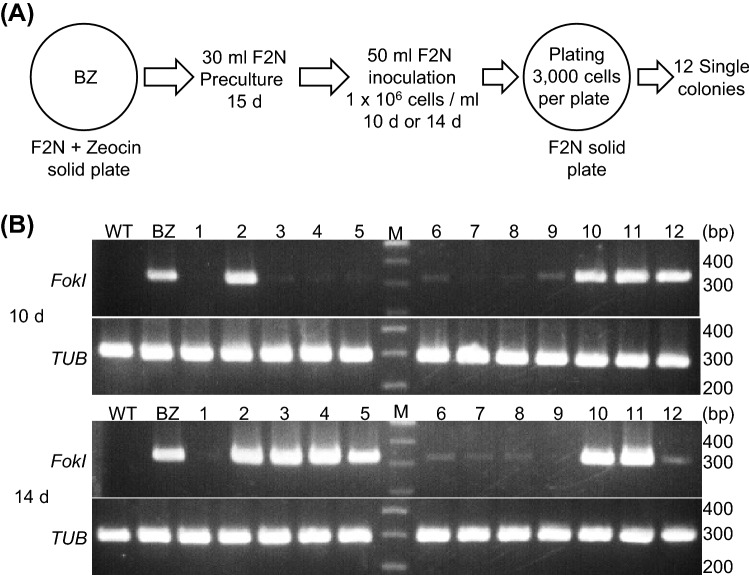
Verification of the efficient clearance method for all-in-one PtTALEN-ARS plasmids from *Nannochloropsis oceanica* cells using solid F2N plates. (**A**) Workflow of the clearance of all-in-one PtTALEN-ARS plasmids from host cells using culture spreading onto solid F2N plates. All-in-one PtTALEN-ARS plasmids could be removed earlier than the procedure shown in the Fig. 2A workflow. (**B**) Verification of the clearance of all-in-one PtTALEN-ARS plasmids from host cells using culture spreading onto solid F2N plates by PCR. The 12 colonies that emerged in F2N without zeocin plates were collected, cultured with 2 mL F2N liquid medium, and used for the PCR analysis. WT: total DNAs extracted from wild-type cells; BZ: total DNAs extracted from individual colonies of all-in-one *NoNR* PtTALEN-ARS plasmids; 1–12: total DNAs extracted from strains collected from F2N solid plates; *FokI*: *FokI* PCR amplicons; *TUB*: *tubulin-beta* PCR amplicons; M: DNA ladder marker. 10 d and 14 d indicate the duration (10 or 14 days) of liquid culture.

To efficiently remove the vectors, we verified the clearance method of all-in-one PtTALEN-ARS vectors. We postulated that the all-in-one PtTALEN-ARS vectors could be removed by a failure of inheritance at cell division or by endogenous degradation. First, we utilized liquid culture with large volumes (30 mL or 25 mL) of F2N medium, as shown in Supplementary Fig. S4A, expecting that the vectors would be eliminated by high cell division. The cells were collected from the large volume liquid F2N culture and confirmed plasmid clearance by PCR analysis (Supplementary Fig. S4B). However, in all tested cells, PCR bands were detected. Next, we attempted to cultivate strains harboring the all-in-one PtTALEN-ARS plasmids under phosphorus or nitrogen deficiency, as shown in Supplementary Fig. S5A, since these stress conditions may stimulate various endogenous degradation systems in *Nannochloropsis* species^25^. Total DNAs were extracted from cells and vector clearance was confirmed by PCR (Supplementary Fig. S5B). The results showed that PCR bands were also detected in all tested conditions. Finally, we attempted to isolate single cells using solid F2N plates, as shown in Fig. 4A, since it was judged that it would be difficult to completely eliminate cells harboring vectors from the cell population in which vectors were removed. Transformants were cultured in F2N liquid medium for 10 or 14 days and the resultant cultures were plated onto F2N solid plates. Single colonies were collected and total DNAs extracted from these colonies were used for PCR analysis (Fig. 4B). The results showed that PCR band intensities were substantially reduced in 8/12 (10 d) or 6/12 (14 d) of the tested colonies. We found that strain isolation using solid plates was essential for obtaining strains in which plasmids were removed.

## Discussion

We constructed all-in-one PtTALEN-ARS plasmids, which have yeast CEN/ARS and confirmed that high genome editing activity was maintained (Fig. 1). We also confirmed the removability for these plasmids (Figs. 2 and 3). In the PCR analysis of plasmid clearance, a trance amount of PCR band was detected in some cell lines (Figs. 2B and 4B). The copy number of plasmids containing CEN/ARS in *Nannochloropsis* species was not studied. However, it is unlikely that the copy number of all-in-one PtTALEN-ARS plasmids fluctuated in *Nannochloropsis* cells, because the copy numbers of plasmids containing CEN/ARS of less than ten in yeast^26^ or diatoms^16^ were previously reported. To elucidate this, additional quantitative analysis is necessary, but we believe that the trace amount of PCR bands may reflect the ratio of quite few population of cells harboring plasmids to total cell population. In addition, the effect of episomal vectors on *Nannochloropsis* cells growth and lipid accumulation was checked. In the growth analysis of tested strains, no significant difference was detected (Supplementary Fig. S3A). On the other hand, one of the genome-edited strain removed episomal vectors (Strain ID: A5) accumulated significantly higher TAG contents than the parental strain (Strain ID: AZ) which still harbors the episomal vectors (Supplementary Fig. S3B). A significant difference in TAG contents among the other tested strains was not found. Because TAG over-accumulation was not detected in B1 strain even though both A5 (Δ11) and B1 (Δ22) strains have a frame shift mutation in nitrate reductase locus, it is not likely that TAG contents were enhanced by nitrate reductase mutation and reduced by episomal vector harboring in the AZ strain. We concluded that the effect of harboring episomal vector for lipid accumulation was not detected reproducibility. Even if some genome-editing tools containing episomal vectors or vector itself had some inconvenient effects for host cells, these vectors can be removed. Therefore, vector removability is a strong advantage for a molecular breeding tool. Furthermore, we successfully performed carrier DNA-free electroporation using an Elepo21 electroporator (Supplementary Table S3). Because carrier-DNAs may not be able to remove after transformation, carrier-DNA-free electroporation is also an indispensable technique for a transgene-free genome editing system. To remove all-in-one PtTALEN-ARS plasmids efficiently, we employed large volume F2N liquid culture (Supplementary Fig. S4), since we postulated that the proportion of cells with plasmid removal due to failed plasmid inheritance would be elevated by increasing the number of host cell divisions. We also assumed that all-in-one PtTALEN-ARS plasmids might be degraded by an endogenous degradation system under stress conditions, since all-in-one PtTALEN-ARS vectors are not necessary for the survival of algal cells. We attempted to stimulate the degradation of all-in-one PtTALEN-ARS vectors by culturing under phosphorus or nitrogen starvation conditions (Supplementary Fig. S5). However, this mechanism of plasmid clearance was not verified. On the other hand, plasmid-eliminated strains could be collected from isolated colonies using F2N solid plates (Fig. 4). This is consistent with a previous report demonstrating that CRISPR-Cas9 all-in-one vectors containing CEN/ARS could be eliminated by single colony isolation using solid F2N plates^9^. In accordance with these findings, we conclude that single colony isolation using solid plates is essential for the clearance of all-in-one PtTALEN-ARS plasmids.

Verruto et al*.* reported an endogenous gene disruption system involving the insertion of an antibiotic marker gene via CRISPR-Cas9 and subsequent marker gene removal using Cre recombinase in *Nannochloropsis* species^10^. Furthermore, a septuple gene disruption mutant was constructed by serial use of this system. However, after marker removal by Cre recombinase, the lox sequence, which is regarded as a transgene, is retained at the insertion locus. Naduthodi et al*.* reported the use of ribonucleoprotein (RNP) consisting of the purified Cas9 proteins and in vitro synthesized sgRNAs in *Nannochloropsis* species^8^. In this system, the antibiotic gene fragments are indispensable for transformant selection. Recently, application of CRISPR-Cas9 RNP systems applied to microalgae was reported^8,27–29^. In these systems, the selection of transformed cells can be a hurdle because the transformation efficiency is generally low. Therefore, a selection marker that is also regarded as a transgene or some other selection system is required. Poliner et al*.* reported that CRISPR-Cas9 all-in-one vectors containing CEN/ARS behaved as episomal plasmids and could be removed from *Nannochloropsis* species cells^9^. They constructed an efficient vector removal system that relied on salmon sperm DNA, which is regarded as a transgene source, for electroporation. The CRISPR-Cas9 system has an ease of use advantage; however, its targeting is limited by its requirement for specific PAM sequences. Furthermore, there is a risk that the transgenes remain and disrupt host cell chromosomes, due to insertion of the reverse-transcribed sgRNAs of CRISPR-Cas9. Ono et al*.* reported the insertion of reverse-transcribed sgRNAs at the target site of Cas9 in mouse zygotes^30^. Therefore, even if a genome-edited strain is generated using RNPs or CRISPR-Cas9 plasmids containing CEN/ARS, verification of sgRNA insertion is indispensable for the construction of transgene-free strains.

This new removable genome editing tool is advantageous for generating transgene-free high-performance algae or multi-deletion mutants, since the all-in-one PtTALEN-ARS plasmids containing antibiotic markers are not inserted into host chromosomes. This is also convenient for eliminating the possibility of disrupting endogenous genes due to plasmid insertion. Furthermore, transgene-free genome edited algae can be produced by introducing the removable all-in-one PtTALEN-ARS plasmids into *N. oceanica* cells using the carrier DNA-free electroporation system. We anticipate that our system will become an essential technology for constructing feasible high-performance algal strains that are adapted to outdoor culture for biodiesel production.

## Methods

### Materials and culture conditions

*Nannochloropsis oceanica* NIES-2145 was obtained from the National Institute for Environmental Studies in Japan (NIES). *N. oceanica* NIES-2145 was grown in F2N medium under continuous light (50 µmol of photons m^−2^ s^−1^) at 25 °C. F2N medium was prepared as described previously^20^. F2N 50% seawater medium plates containing 2 µg L^−1^ zeocin (Nacalai Tesque) was used for selection of transformants. pHSN401 and pRS314-URA3 were obtained from Addgene.

### Construction of all-in-one PtTALEN-ARS plasmids

The design and insertion of DNA binding motifs for *NoNR* into Nanno-47-TALEN, and codon optimization of Platinum TALEN for *N. oceanica* were described in a previous report^17^. pMD20-TALEN-47-ZeoR and pMD20-BamHI-TALEN47 were constructed as previously described^17^. The right TALEN expression plasmids, containing a kanamycin resistance marker, were constructed upon inserting an expression cassette for the kanamycin resistance gene and removing the ampicillin resistance gene as follows. The kanamycin expression cassette (fragment #1) was amplified with pHSN401 as a template and KanR-ins-F and KanR-ins-R primers. The PCR product (fragment #2) was amplified with pMD20-BamHI-TALEN47 as a template and the Am-delta-F and KanR-clone-R primers. The PCR product (fragment #3) was amplified with pMD20-BamHI-TALEN47 and the Marker-sequence-F and Am-delta-R primers. pMD20-TALEN-KanR-BsmBI was constructed by integrating fragments #1–3 using an In-Fusion HD cloning kit (Clontech). The *Bsm*BI site of KanR was removed using inverse primer PCR with KanR-dBsmBI-F and KanR-dBsmBI-R primers. pMD20-TALEN-KanR was constructed by self-ligation of the resultant PCR products using an In-Fusion HD cloning kit. ARS (fragment #4) was amplified with pRS314-URA3 as a template and the ARS-ins-1-F and ARS-ins-1-R primers. The PCR product (fragment #5) was amplified with pMD20-TALEN47 as a template and the ARS-cloning-1-F and Marker-cloning-R primers. The PCR product (fragment #6) was amplified with pMD20-TALEN-47-ZeoR and the Ins-Marker-F and ARS-cloning-1-R primers. pMD20-TALEN-ARS-ZeoR was constructed by integrating fragments #4-6. The sequences of primers used for plasmid construction are shown in Supplementary Table S1.

To construct all-in-one PtTALEN-ARS plasmids, the right TALEN expression cassette containing kanamycin resistance gene of pMD20-TALEN-KanR and DNA binding modules for right TALEN were inserted into pMD20-TALEN-ARS-ZeoR containing DNA binding modules for left TALEN using the *Bam*HI and *Eco*RI restriction enzyme sites.

### Treatment and verification of plasmid clearance

The workflow is shown in Fig. 2A. Colonies emerged on F2N 50% seawater containing 2 µg L^−1^ zeocin were inoculated into a 96-well plate with 200 µL of F2N 50% seawater containing 2 µg L^−1^ zeocin and cultured for 1 month. *NoNR* locus genotypes of these strains were checked by direct sequencing as previously reported^17^. The isolated two frame shift mutants, AZ and BZ, were inoculated into an F2N 50% seawater containing 2 µg L^−1^ Zeocin solid plate as a master plate. To remove the all-in-one PtTALEN-ARS plasmids, AZ and BZ, were collected from same 96-well plate and were streaked on an F2N solid plate without zeocin for 1 month. The streaked strains were collected and streaked again on a new F2N solid plate without zeocin for 3 weeks. The two times-streaked strains were inoculated into a 12-well plate with 2 mL of F2N medium without zeocin and cultured statically for 9 days. The liquid F2N cultured strains were collected and named AL or BL. Liquid cultures of AL or BL containing 3000 cells were spread on an F2N plate without zeocin and cultured for 1 month. Colonies that emerged in the plate were collected and used for the following studies as a potential plasmid removed strains (Strain ID: A1-A12 and B1-B12).

The method of efficient plasmid removal is described here. The workflow is shown in Fig. 4A. Transformants of the all-in-one PtTALEN-ARS plasmid were inoculated in 30 mL of F2N liquid medium without zeocin as a preculture for 15 days. Resultant cells were inoculated in 50 mL of F2N liquid medium without zeocin for use as a main culture, adjusting the final cell density to 1 × 10^6^ cells mL^−1^, and cultivated for 10 or 14 days. Resultant cell cultures containing 3000 cells were spread on an F2N solid plate (9 cm diameter) without zeocin. Colonies that emerged in these plates were collected and used for the following studies.

The collected cells were cultured using 12-well plates with 2 mL of F2N liquid medium. Total DNAs from cultured cells were extracted. These total DNAs were used as a template for PCR analysis to confirm plasmid clearance. PCR was performed with KOD-FX-Neo polymerase (TOYOBO) according to the supplier’s protocol. The FokI nuclease domain was amplified using the FokI-F and FokI-R primers. The TALEN N-terminal domain was amplified using the TALEN47-N-F and TALEN47-N-R primers. The kanamycin resistance gene was amplified using the KanR-F and KanR-R primers. The zeocin resistance gene was amplified using the ZeoR-F and ZeoR-R primers. The tubulin-beta gene was amplified using the TUB-F and TUB-R primers. The sequences of these primers are shown in Supplementary Table S1. Uncropped gel images are shown in Supplementary Figs. S6–S12.

### Total DNA extraction, HMA, Cel-I assay, and direct sequencing

Introduction of all-in-one TALEN-ARS plasmids into algal cells was performed by using Gene pulser II (Bio-Rad) as described by Killian et al.^21^. Total genomic DNA extraction was performed as described in a previous report^17^. HMAs, Cel-I assays, and direct sequencing were performed as described previously^17^.

### Electroporation using an Elepo21 electroporator

*Nannochloropsis* cells were cultured using 50 mL of F2N medium for 10 days as a preculture. Then, 6.25 × 10^8^ cells of precultured cells were inoculated into 250 mL of F2N and were cultured for 4 days. Cultured cells were washed twice with 150 mL of ice cold 375 mM d-sorbitol. The cells were then washed twice with 80 mL of ice-cold 375 mM d-sorbitol. The concentration of washed cells was adjusted to 1 × 10^10^ cells mL^−1^ in d-sorbitol. Then, 38 µL of cells and 400 ng of DNA of all-in-one PtTALEN-ARS targeting *NoNR* were mixed and placed into the 1 mm gap of an electroporation cuvette (NEPAGENE). The cuvettes containing both cells and DNA were incubated at room temperature for 10 min. The cuvettes were then incubated on ice for 5 min. Electroporation was performed using an Elepo21 electroporator (NEPAGENE). The electroporation settings are shown in Supplementary Table S2. The cells were quickly suspended in 5 mL of F2N liquid medium in a 15 mL plastic tube immediately after electroporating. Plastic tubes containing electroporated cells were cultured for 2 days under dim light conditions by wrapping the plastic tube in a paper towel. The resultant cells were collected by centrifugation and resuspended in 2 mL of melted F2N medium containing 0.4% agar (top agar). The cells suspended in top agar were then plated on a F2N 50% seawater medium plate containing 2 µg L^−1^ of zeocin (Nacalai Tesque). The plates were subsequently cultured for about 1 month.

### Spot test

Spot tests were performed as in a previous report^17^ using solid plates of F2N, F2N 50% seawater containing 2 µg L^−1^ zeocin (F2N + Zeo), and F2N without ammonium (F2N–NH_4_^+^).

### Lipid extraction, lipid separation, and GC analysis

Total lipid extraction from *Nannochloropsis* cells was performed as previously reported^22–24,31^. Lipids were separated by using thin-layer chromatography (TLC). TLC was developed using hexane/diethyl ether/acetic acid (40:10:1, v/v/v). Lipids were visualized by 0.01% (w/v) primuline in 80% (v/v) acetone under UV light. TAG spots were scraped off the TLC plates, and fatty acids of TAGs were converted into fatty acid methyl esters (FAMEs), as previously described^22–24,31^. The qualitative composition of FAMEs was determined by GC-2014 (Shimadzu Corporation) using an internal standard, heneicosylic acid (21:0). GC analysis was performed as previously reported^22–24,31^.

## Supplementary Information

Supplementary Legends.

Supplementary Figures.

Supplementary Table S1.

Supplementary Table S2.

Supplementary Table S3.
