# Supplementary material for: Genome editing with removable TALEN vectors harboring a yeast centromere and autonomous replication sequence in oleaginous microalga
Source: Sci Rep. 2022 Feb 15;12:2480. doi: 10.1038/s41598-022-06495-y (PMC8847555; doi:10.1038/s41598-022-06495-y)
Supplement: Supplementary file 1 — Supplementary Legends. [file 41598_2022_6495_MOESM1_ESM.docx]

**Supplementary information**

**Supplementary Table S1 Primers used in this study.**

**Supplementary Table S2 Electroporation settings used with the Elepo21 electroporator for *Nannochloropsis*.**

**Supplementary Table S3 Carrier DNA-free electroporation using an Elepo21 electroporator in *Nannochloropsis*.**

**Supplementary Figure S1 Workflow for the construction of all-in-one PtTALEN plasmids containing two antibiotic markers.**

Schematic flow of the construction of the TALEN plasmids containing two antibiotic markers for algal expression is shown. ProLHC: LHC promoter; terFCP: FCP terminator; AmpR: Ampicillin resistance gene; KanR: Kanamycin resistance gene; Sh ble: Zeocin resistance gene; E-TALEN: Empty TALEN; L-TALEN: Left-TALEN; R-TALEN: Right-TALEN.

**Supplementary Figure S2 Construction of all-in-one PtTALEN plasmids containing two antibiotic markers.**

(A) Verification of plasmid construction by colony PCR. Colony PCR was performed using SapphireAmp Fast PCR Master Mix (Takara) with the KanR-sequence-F and Marker sequence-R primers. The sequences of these primers are shown in Table S1. 1-8: colonies emerged in LB plates containing ampicillin and kanamycin; M: DNA ladder marker. Arrowhead indicates the PCR amplicons. (B) Verification of vector construction by restriction enzyme digestion with *Bam*HI and *Eco*RI. 1-3: plasmids extracted from colonies emerged on LB plates containing ampicillin and kanamycin; M: DNA ladder marker. Arrowheads indicate the cleaved bands.

**Supplementary Figure S3 Verification of the effect of episomal vector transformation on cell growth and lipid accumulation.**

(A) Growth curve of nitrate reductase genome-edited strains with (Strain ID: AZ or BZ) or without (Strain ID: A5 or B1) episomal vectors. These strains were cultured with 50 mL of F2N liquid medium, and the cell concentration was measured using a hemocytometer. WT: Wild type of *N. oceanica* cells; AZ and BZ: a single colony of *N. oceanica* that emerged in a selectable plate by the introduction of all-in-one *NoNR* PtTALEN-ARS plasmids; A5 and B1: the strains of all-in-one *NoNR* PtTALEN-ARS plasmids removed from AZ or BZ. AZ and A5 are Δ22 *NoNR* frame shift mutants and BZ and B1 are Δ11 *NoNR* frame shift mutants. Data represent means ± standard errors (n = 3). The significant differences among strains were tested using Tukey’s multiple comparison test at every time point. No significant difference was detected. (B) Triacylglycerol (TAG) contents per mL culture or cell. Each strain was cultured with 50 mL of F2N liquid medium without zeocin for 13 days and used for the following lipid analysis. Data represent means ± standard errors (n = 3). Significant differences among strains were tested using Tukey’s multiple comparison test. **P* < 0.05; ***P* < 0.01; the other differences are not significant. Tukey’s multiple comparison test was performed using software, R version 3.2.2 (2015-08-14). (R Core Team (2015). R: A language and environment for statistical computing. R Foundation for Statistical Computing, Vienna, Austria. URL https://www.R-project.org/.

**Supplementary Figure S4 Verification of all-in-one PtTALEN-ARS plasmid clearance by large volume F2N liquid culture.**

(A) Workflow of all-in-one PtTALEN-ARS plasmid clearance from host cells using large volume liquid culture. (B) PCR verification of the clearance of all-in-one PtTALEN-ARS plasmids from host cells cultured in large volume liquid F2N medium. *FokI*: *FokI* PCR amplicons; *TUB*: *tubulin-beta* PCR amplicons; M: DNA ladder marker; WT: total DNAs form wild type cells; AZ and BZ: total DNAs form single colonies emerging following introduction of all-in-one *NoNR* PtTALEN-ARS plasmids. The duration of preculture (4 d or 15 d) is indicated above the line. The duration of liquid culture (7 d, 10 d or 14 d) is indicated below the line.

**Supplementary Figure S5 Verification of all-in-one PtTALEN-ARS plasmid clearance by F2N liquid culture lacking sources of phosphorus or nitrogen.**

(A) Workflow of all-in-one PtTALEN-ARS plasmid clearance from host cells using F2N liquid culture lacking nitrogen or phosphorus sources. (B) PCR verification of the clearance of all-in-one PtTALEN-ARS plasmids from host cells cultured with liquid F2N medium lacking phosphorus or nitrogen sources. *FokI*: *FokI* PCR amplicons; *TUB*: *tubulin-beta* PCR amplicons; WT: total DNAs form wild type cells; BZ: total DNAs form single colonies emerging following introducing of all-in-one *NoNR* TALEN-ARS plasmids; M: DNA ladder marker. F4, P4, N4, F12, P12, and N12 indicate the total DNAs isolated from cells treated with the following medium and duration of liquid culture. F4 and F12: cultivation with F2N medium for 4 days or 12 days; P4 and P12: cultivation with F2N without a phosphorus source for 4 days and 12 days; N4 and N12: cultivation with F2N without a nitrogen source for 4 days and 12 days.

**Supplementary Figure S6 Gel image of electrophoresis.**

Gel image of HMA and Cel-I assay of all-in-one TALEN-ARS plasmid transformants is shown. The right side of this gel image was eliminated because there were unrelated data for this study. The data using a cropped image are shown in Figure 1C.

**Supplementary Figure S7 Full gel images of electrophoresis.**

Full gel images of PCR analysis using *FokI*, or *TUB* primers are shown. The data using cropped images are shown in Figure 2B.

**Supplementary Figure S8 Full gel images of electrophoresis.**

Full gel images of PCR analysis using *TALEN-N*, *FokI*, *KanR*, *ZeoR*, or *TUB* primers are shown. Arrowheads indicate the PCR bands using *TALEN-N* primers. The data using cropped images were shown in Figure 2C.

**Supplementary Figure S9 Full gel images of electrophoresis.**

Full gel images of PCR analysis using *FokI*, or *TUB* primers were shown. The data using cropped images were shown in Figure 4B.

**Supplementary Figure S10 Gel images of electrophoresis.**

(A) Gel image of colony PCR analysis using KanR-sequence-F and Marker sequence-R primers is shown. (B) Gel image of restriction enzyme treatment of all-in-one TALEN plasmids is shown. The right side of these gel images has been eliminated because there were data unrelated to this study. Arrowheads indicate the cleaved bands. The data using cropped images were shown in Supplementary Figs. S2A and S2B.

**Supplementary Figure S11 Full gel images of electrophoresis.**

Full gel images of PCR analysis using *FokI*, or *TUB* primers are shown. The data using cropped images were shown in Supplementary Figure S4B.

**Supplementary Figure S12 Full gel image of electrophoresis.**

Full gel image of PCR analysis using *FokI*, or *TUB* primers is shown. The data using a cropped image is shown in Supplementary Figure S5B.
