# Supplementary material for: Genome editing with removable TALEN vectors harboring a yeast centromere and autonomous replication sequence in oleaginous microalga
Source: Sci Rep. 2022 Feb 15;12:2480. doi: 10.1038/s41598-022-06495-y (PMC8847555; doi:10.1038/s41598-022-06495-y)
Supplement: Supplementary file 2 — Supplementary Figures. [file 41598_2022_6495_MOESM2_ESM.pptx]

## Slide 1
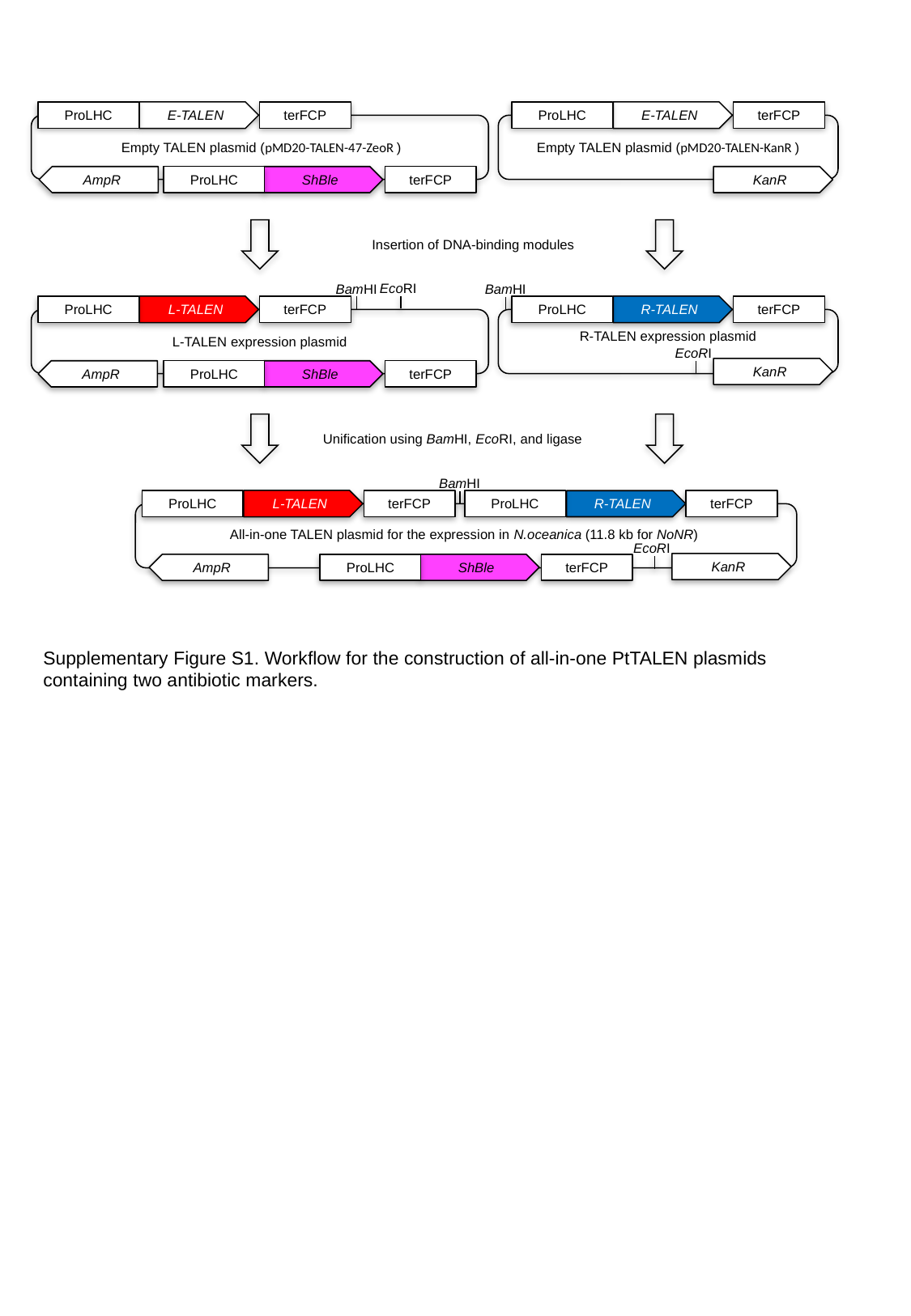

E-TALEN
ProLHC
terFCP
Empty TALEN plasmid (pMD20-TALEN-47-ZeoR )
ProLHC
terFCP
ShBle
AmpR
E-TALEN
ProLHC
terFCP
Empty TALEN plasmid (pMD20-TALEN-KanR )
KanR
Insertion of DNA-binding modules
EcoRI
BamHI
BamHI
L-TALEN
ProLHC
terFCP
R-TALEN
ProLHC
terFCP
R-TALEN expression plasmid
L-TALEN expression plasmid
EcoRI
KanR
ProLHC
terFCP
ShBle
AmpR
Unification using BamHI, EcoRI, and ligase
BamHI
L-TALEN
ProLHC
terFCP
ProLHC
terFCP
R-TALEN
All-in-one TALEN plasmid for the expression in N.oceanica (11.8 kb for NoNR)
EcoRI
KanR
ProLHC
terFCP
AmpR
ShBle
Supplementary Figure S1. Workflow for the construction of all-in-one PtTALEN plasmids containing two antibiotic markers.

## Slide 2
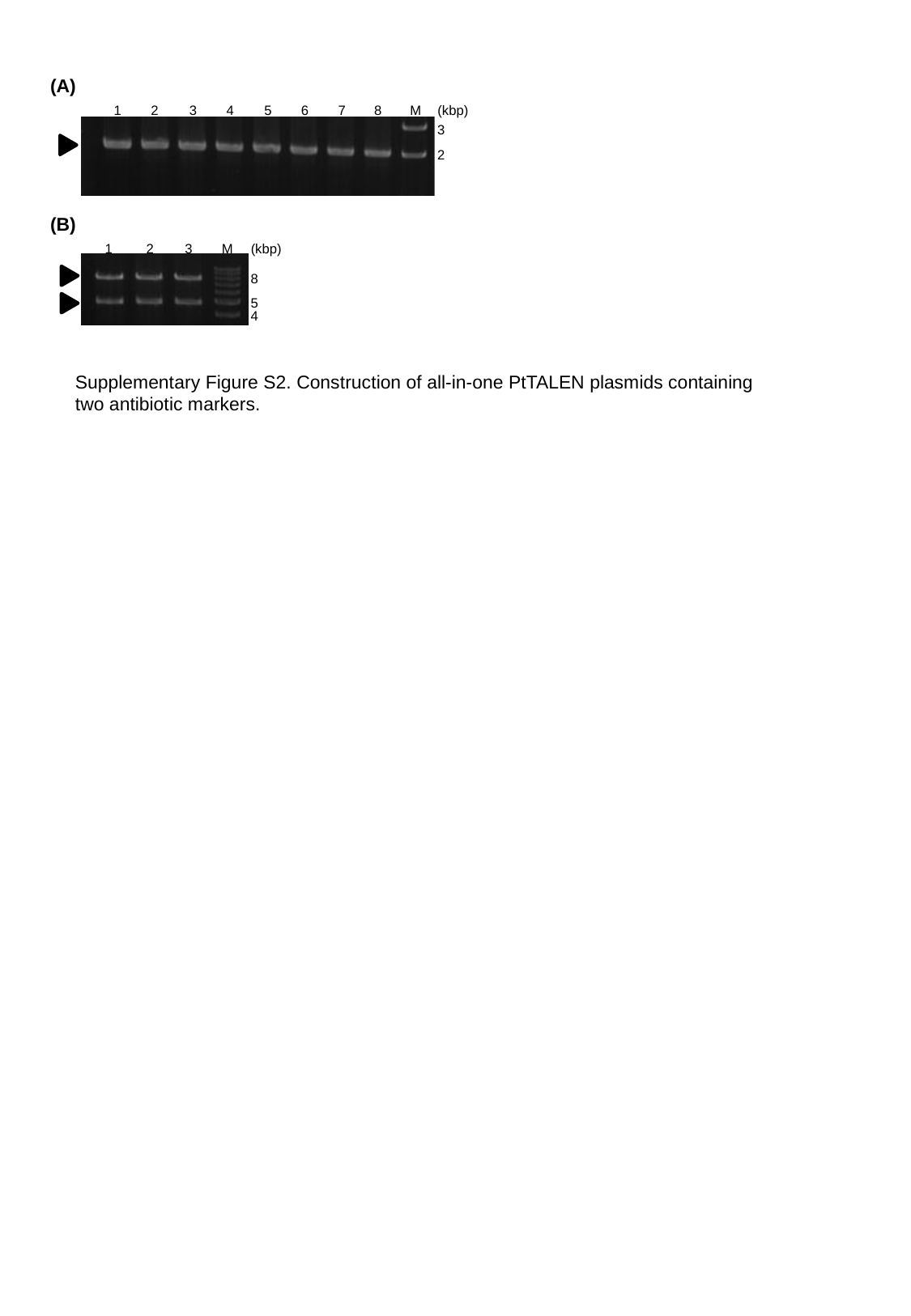

(A)
1
2
3
4
5
6
7
8
M
(kbp)
3
2
(B)
1
2
3
M
(kbp)
8
5
4
Supplementary Figure S2. Construction of all-in-one PtTALEN plasmids containing two antibiotic markers.

## Slide 3
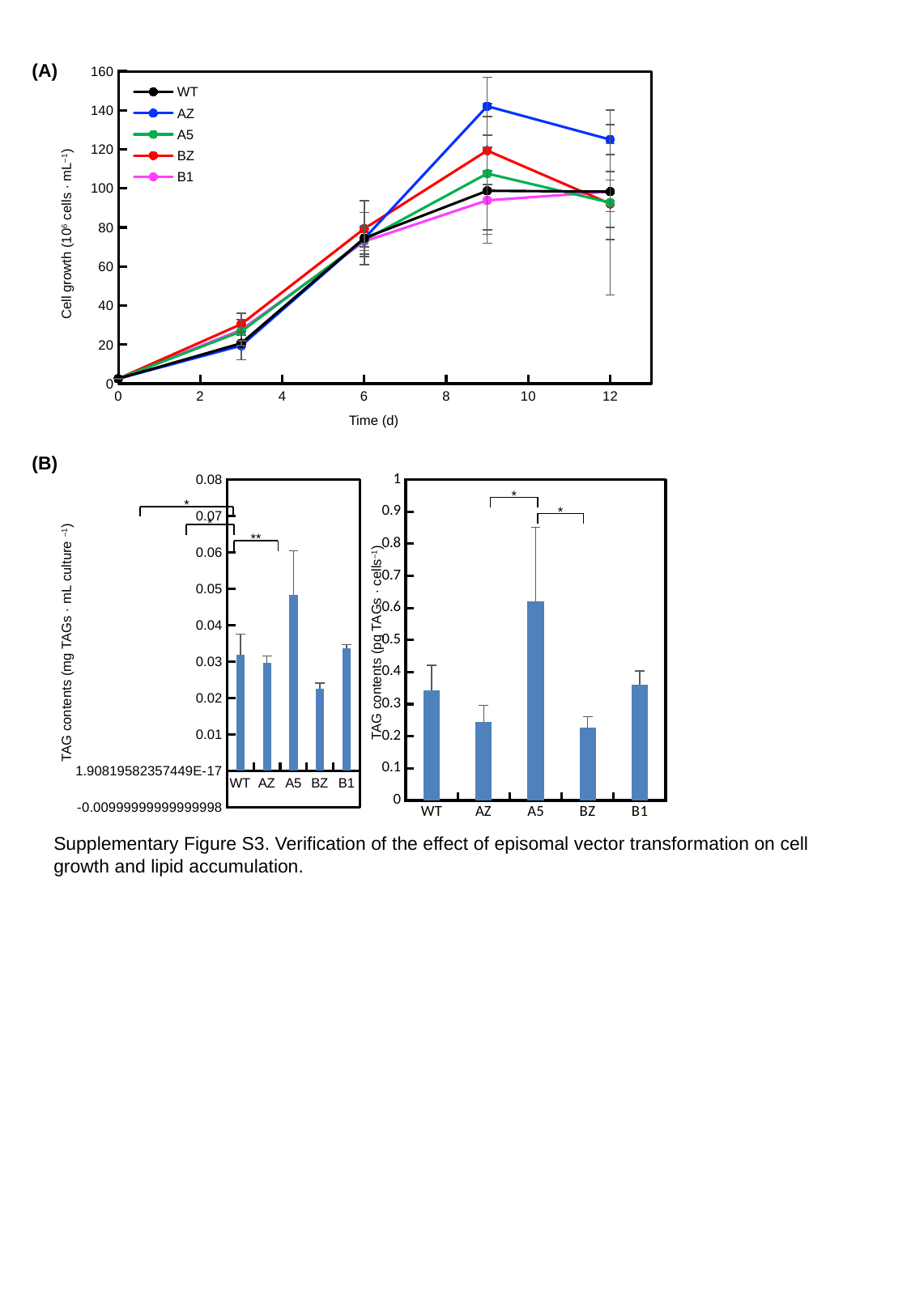

(A)
### Chart
| Category | WT | AZ | A5 | BZ | B1 |
|---|---|---|---|---|---|Cell growth (106 cells ∙ mL–1)
Time (d)
(B)
### Chart
| Category | 培養液あたり |
|---|---|
| WT | 0.03198606801736299 |
| AZ | 0.029683860602915568 |
| A5 | 0.048404977115565136 |
| BZ | 0.022442908054331166 |
| B1 | 0.033573938610633715 |
### Chart
| Category | |
|---|---|
| WT | 0.34191372572300466 |
| AZ | 0.24555581641266291 |
| A5 | 0.6195939175180561 |
| BZ | 0.22712247309613873 |
| B1 | 0.3605038517321426 |*
*
*
*
**
TAG contents (mg TAGs ∙ mL culture –1)
TAG contents (pg TAGs ∙ cells–1)
Supplementary Figure S3. Verification of the effect of episomal vector transformation on cell growth and lipid accumulation.

## Slide 4
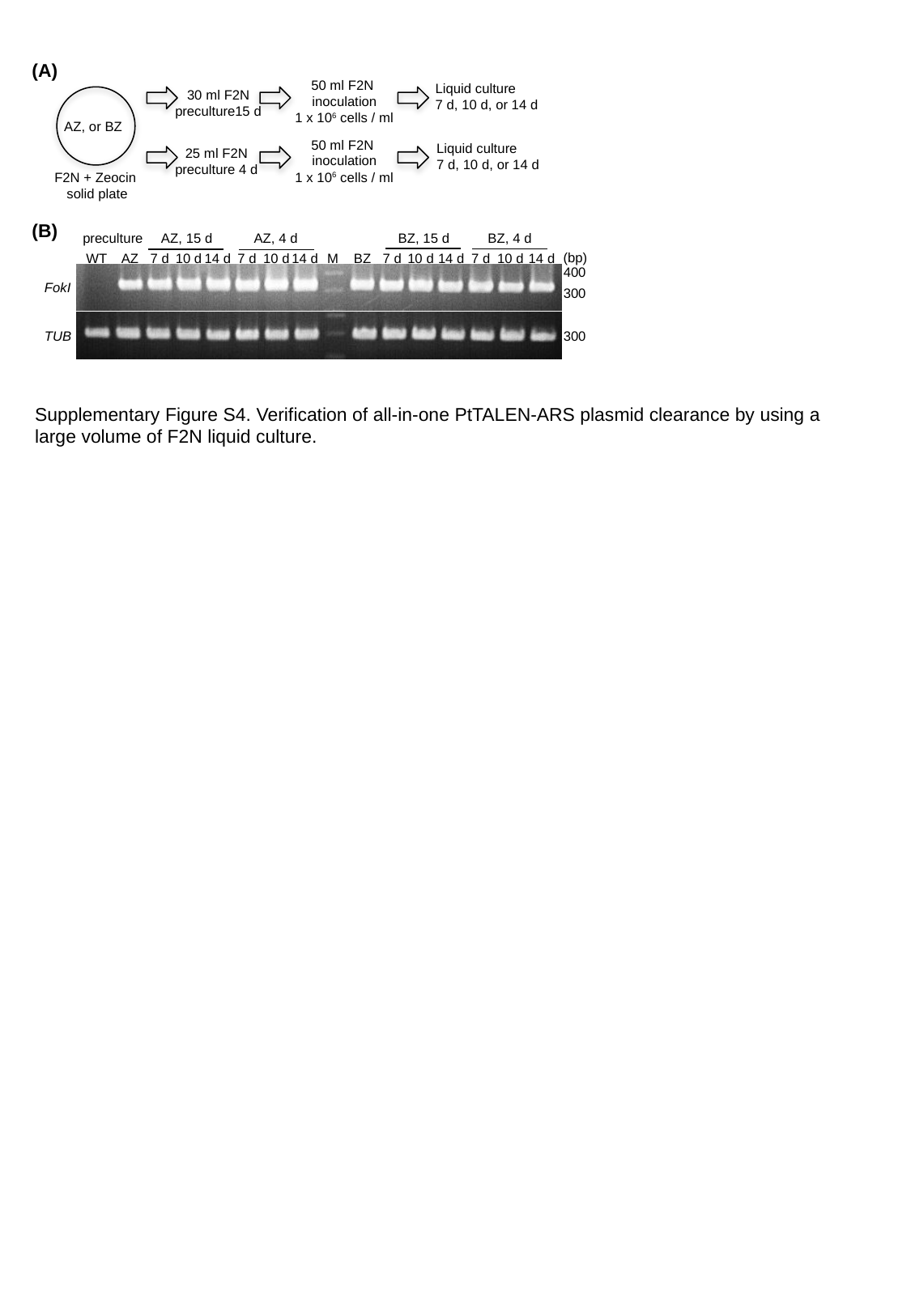

(A)
50 ml F2N
inoculation
1 x 10^6^ cells / ml
50 ml F2N
inoculation
1 x 10^6^ cells / ml
Liquid culture
7 d, 10 d, or 14 d
30 ml F2N
preculture15 d
25 ml F2N
preculture 4 d
AZ, or BZ
Liquid culture
7 d, 10 d, or 14 d
F2N + Zeocin
solid plate
(B)
preculture
AZ, 15 d
AZ, 4 d
BZ, 15 d
BZ, 4 d
(bp)
WT
AZ
7 d
10 d
14 d
7 d
10 d
14 d
M
BZ
7 d
10 d
14 d
7 d
10 d
14 d
400
FokI
300
300
TUB
Supplementary Figure S4. Verification of all-in-one PtTALEN-ARS plasmid clearance by using a large volume of F2N liquid culture.

## Slide 5
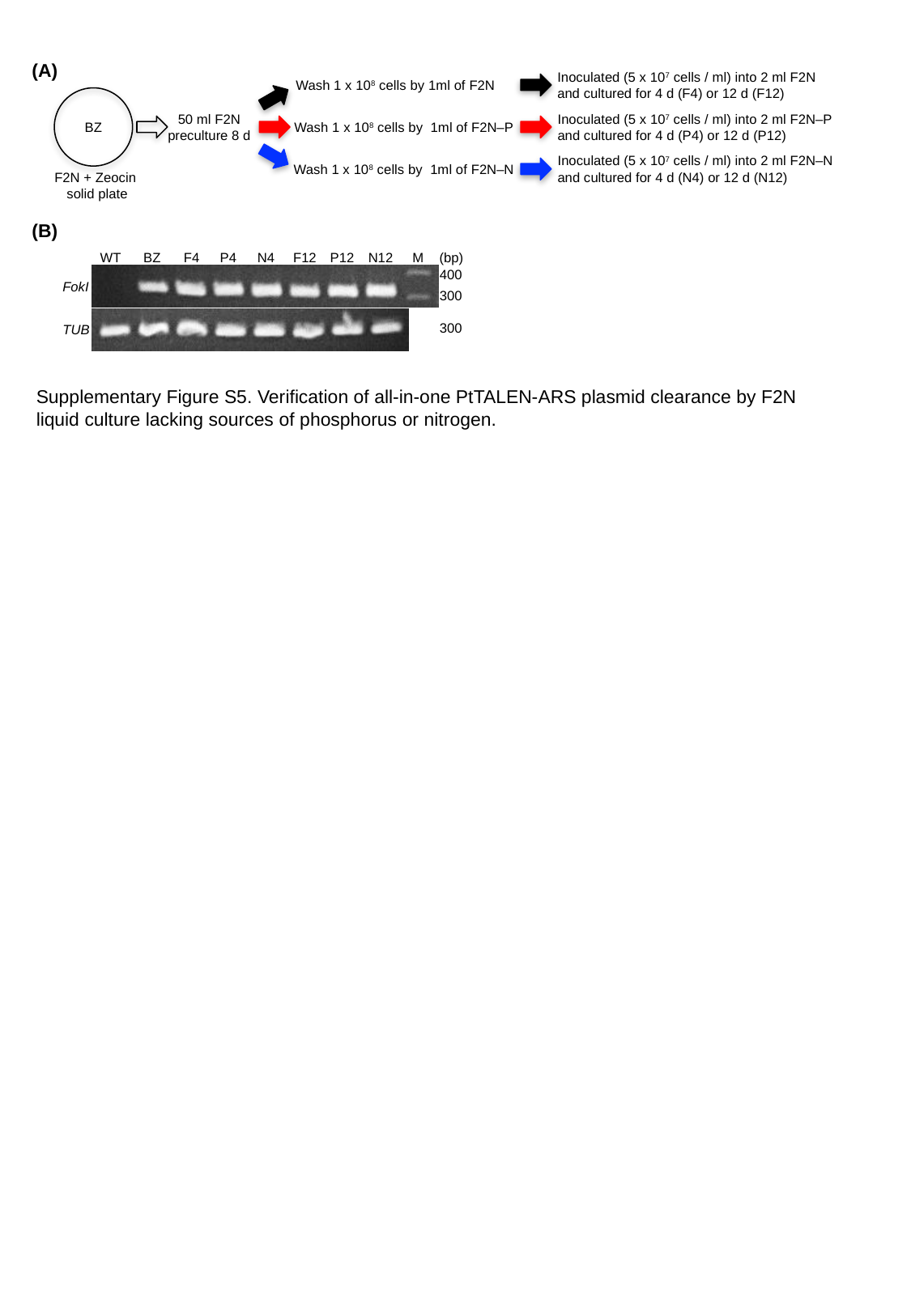

(A)
Inoculated (5 x 10^7^ cells / ml) into 2 ml F2N and cultured for 4 d (F4) or 12 d (F12)
Inoculated (5 x 10^7^ cells / ml) into 2 ml F2N–P and cultured for 4 d (P4) or 12 d (P12)
Inoculated (5 x 10^7^ cells / ml) into 2 ml F2N–N and cultured for 4 d (N4) or 12 d (N12)
Wash 1 x 10^8^ cells by 1ml of F2N
Wash 1 x 10^8^ cells by 1ml of F2N–P
Wash 1 x 10^8^ cells by 1ml of F2N–N
BZ
50 ml F2N
preculture 8 d
F2N + Zeocin
solid plate
(B)
WT
BZ
F4
P4
N4
F12
P12
N12
M
(bp)
400
FokI
300
300
TUB
Supplementary Figure S5. Verification of all-in-one PtTALEN-ARS plasmid clearance by F2N liquid culture lacking sources of phosphorus or nitrogen.

## Slide 6
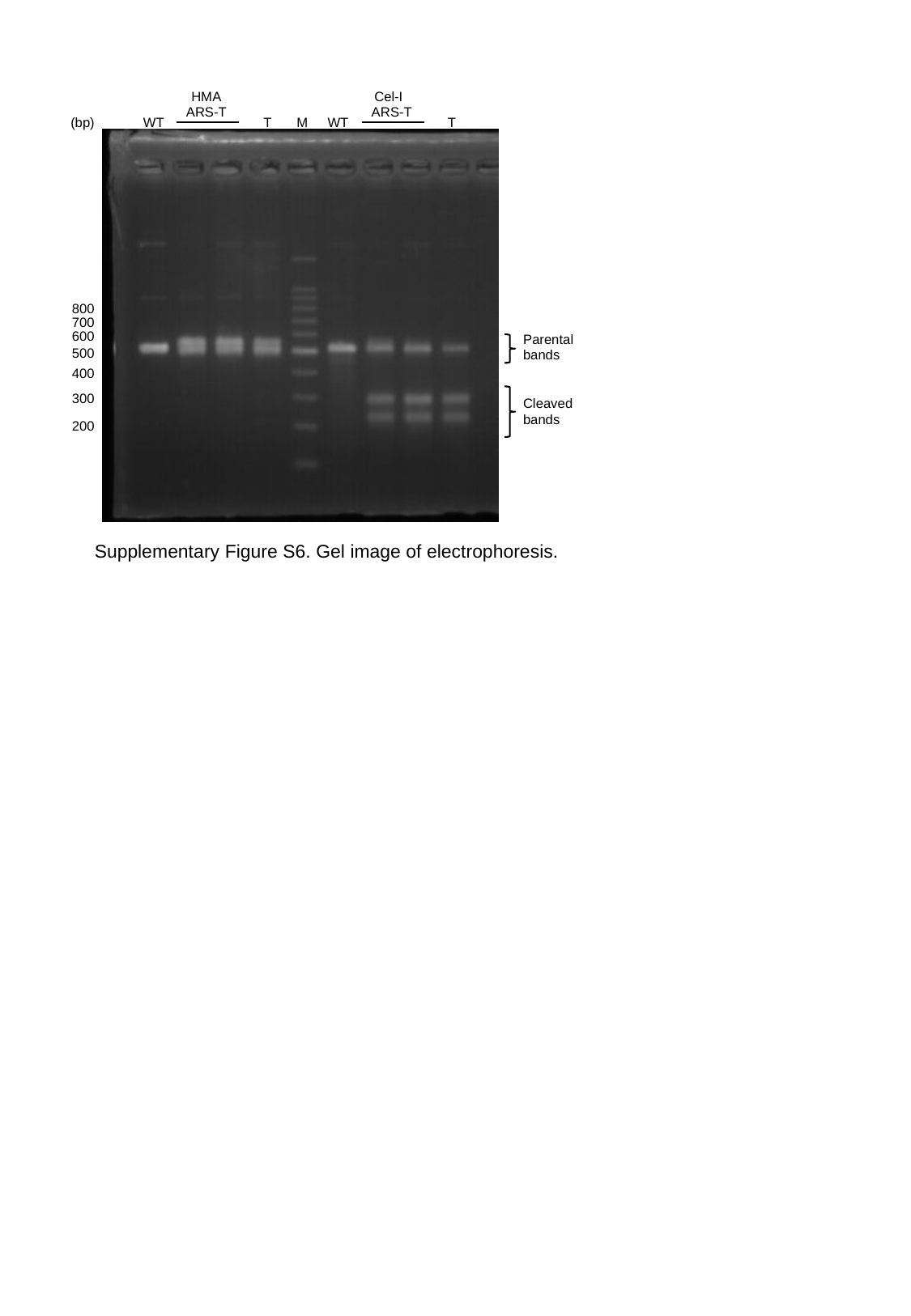

HMA
Cel-I
ARS-T
ARS-T
(bp)
WT
T
M
WT
T
800
700
600
Parental
bands
500
400
300
Cleaved
bands
200
Supplementary Figure S6. Gel image of electrophoresis.

## Slide 7
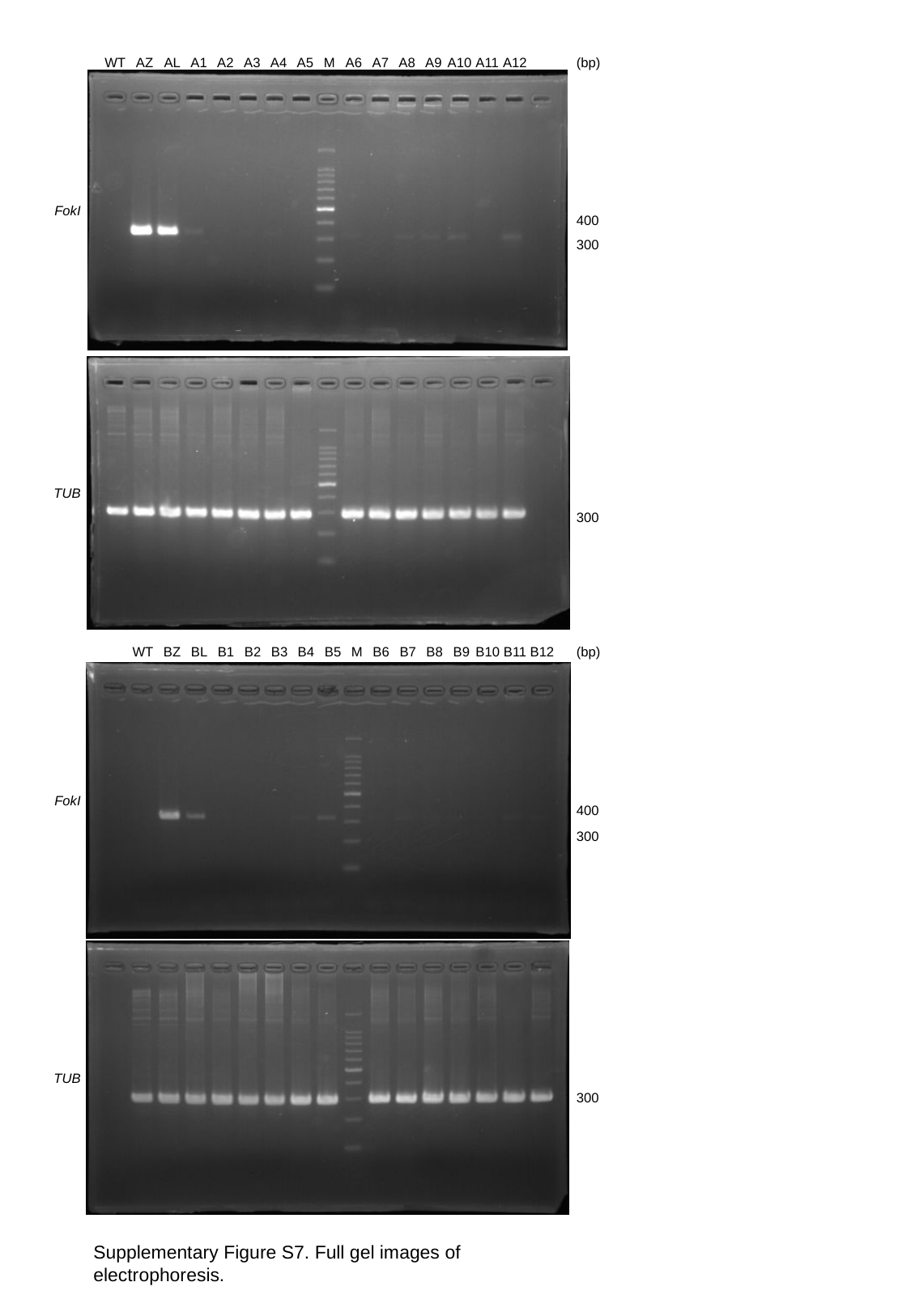

WT
AZ
AL
A1
A2
A3
A4
A5
M
A6
A7
A8
A9
A10
A11
A12
(bp)
FokI
400
300
TUB
300
WT
BZ
BL
B1
B2
B3
B4
B5
M
B6
B7
B8
B9
B10
B11
B12
(bp)
FokI
400
300
TUB
300
Supplementary Figure S7. Full gel images of electrophoresis.

## Slide 8
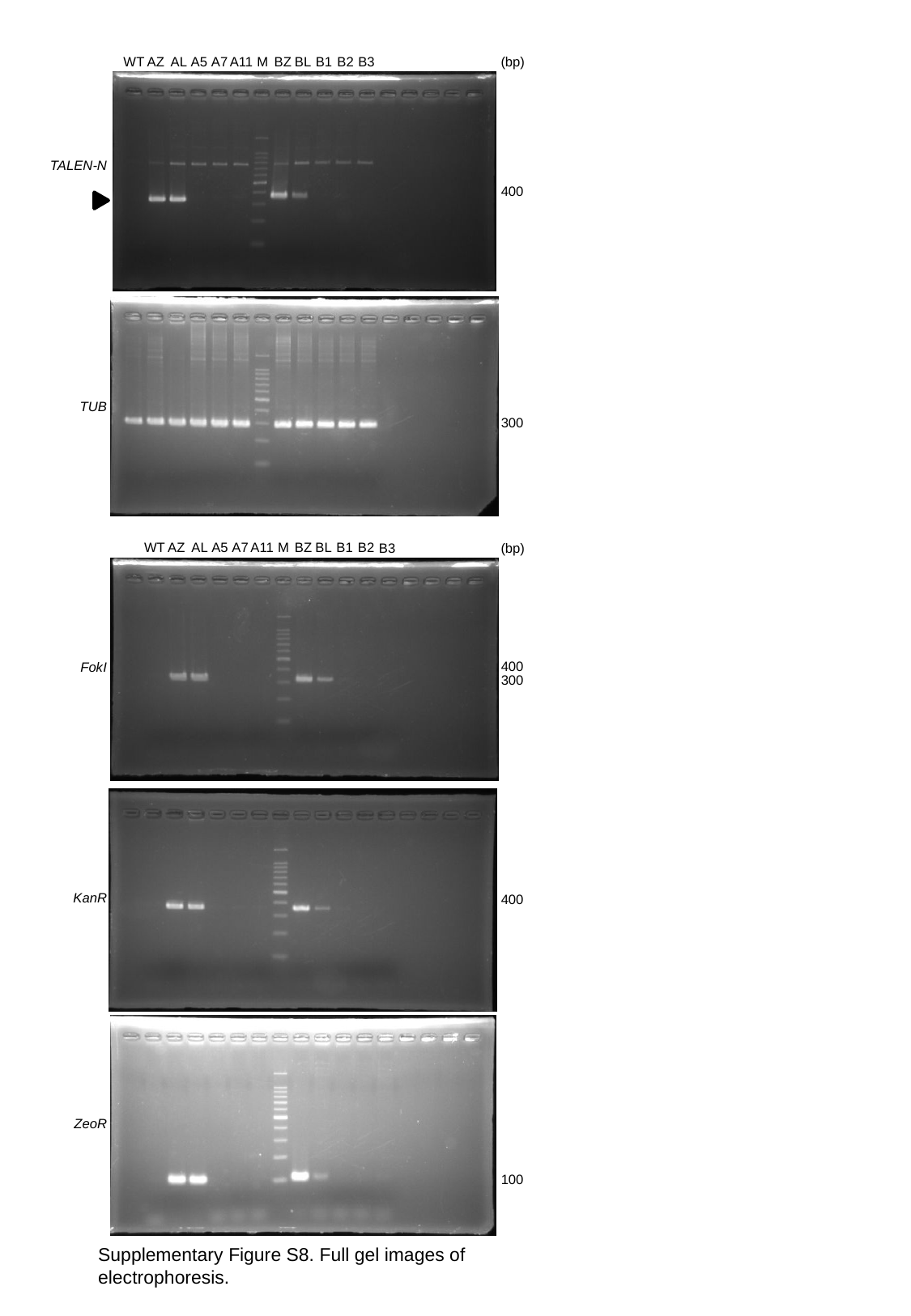

WT
AZ
AL
A5
A7
A11
M
BZ
BL
B1
B2
B3
(bp)
TALEN-N
400
TUB
300
BZ
WT
AZ
AL
A5
A7
B2
A11
M
BL
B1
B3
(bp)
400
FokI
300
KanR
400
ZeoR
100
Supplementary Figure S8. Full gel images of electrophoresis.

## Slide 9
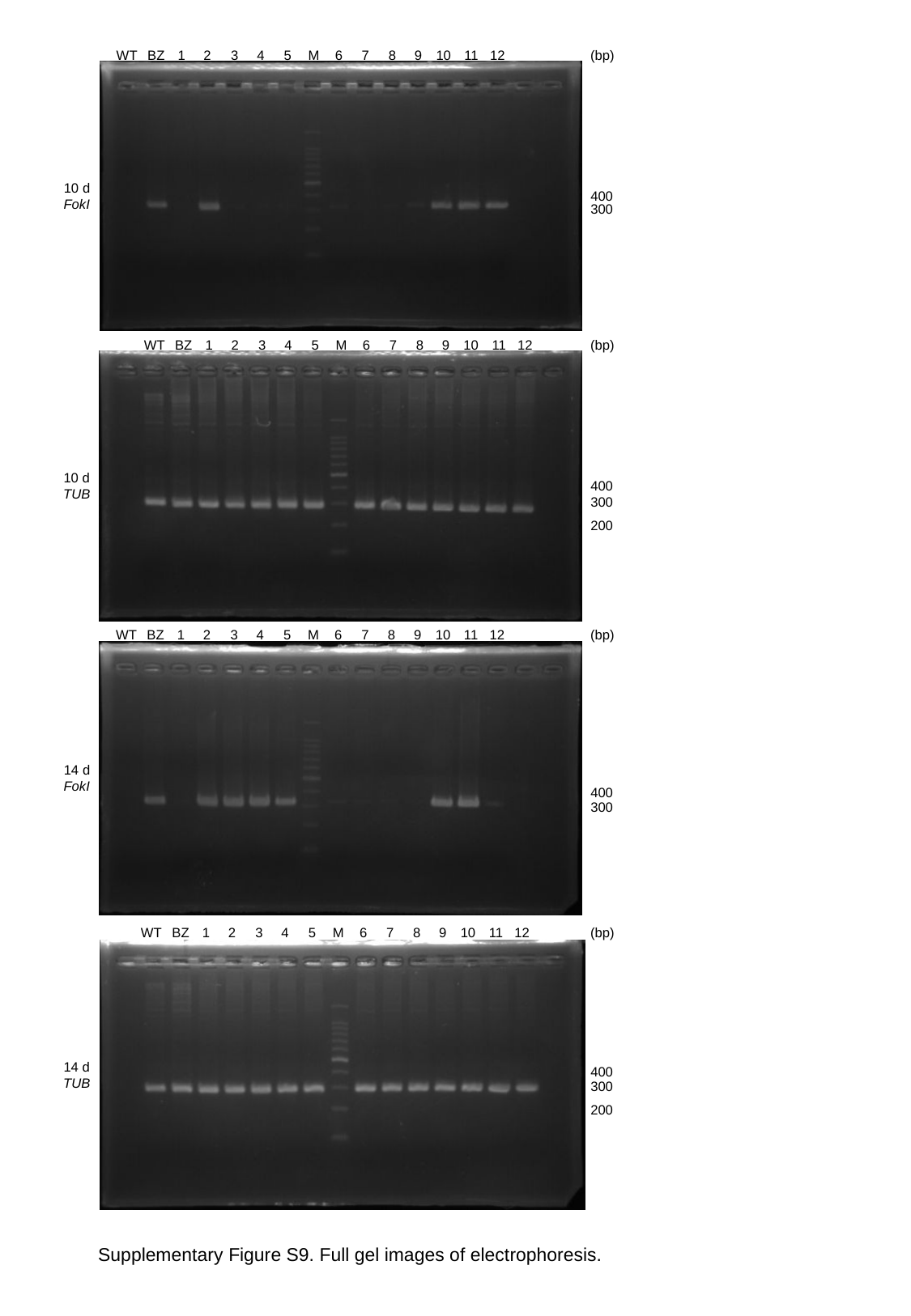

WT
BZ
1
2
3
4
5
M
6
7
8
9
10
11
12
(bp)
10 d
FokI
400
300
WT
BZ
1
2
3
4
5
M
6
7
8
9
10
11
12
(bp)
10 d
TUB
400
300
200
WT
BZ
1
2
3
4
5
M
6
7
8
9
10
11
12
(bp)
14 d
FokI
400
300
WT
BZ
1
2
3
4
5
M
6
7
8
9
10
11
12
(bp)
14 d
TUB
400
300
200
Supplementary Figure S9. Full gel images of electrophoresis.

## Slide 10
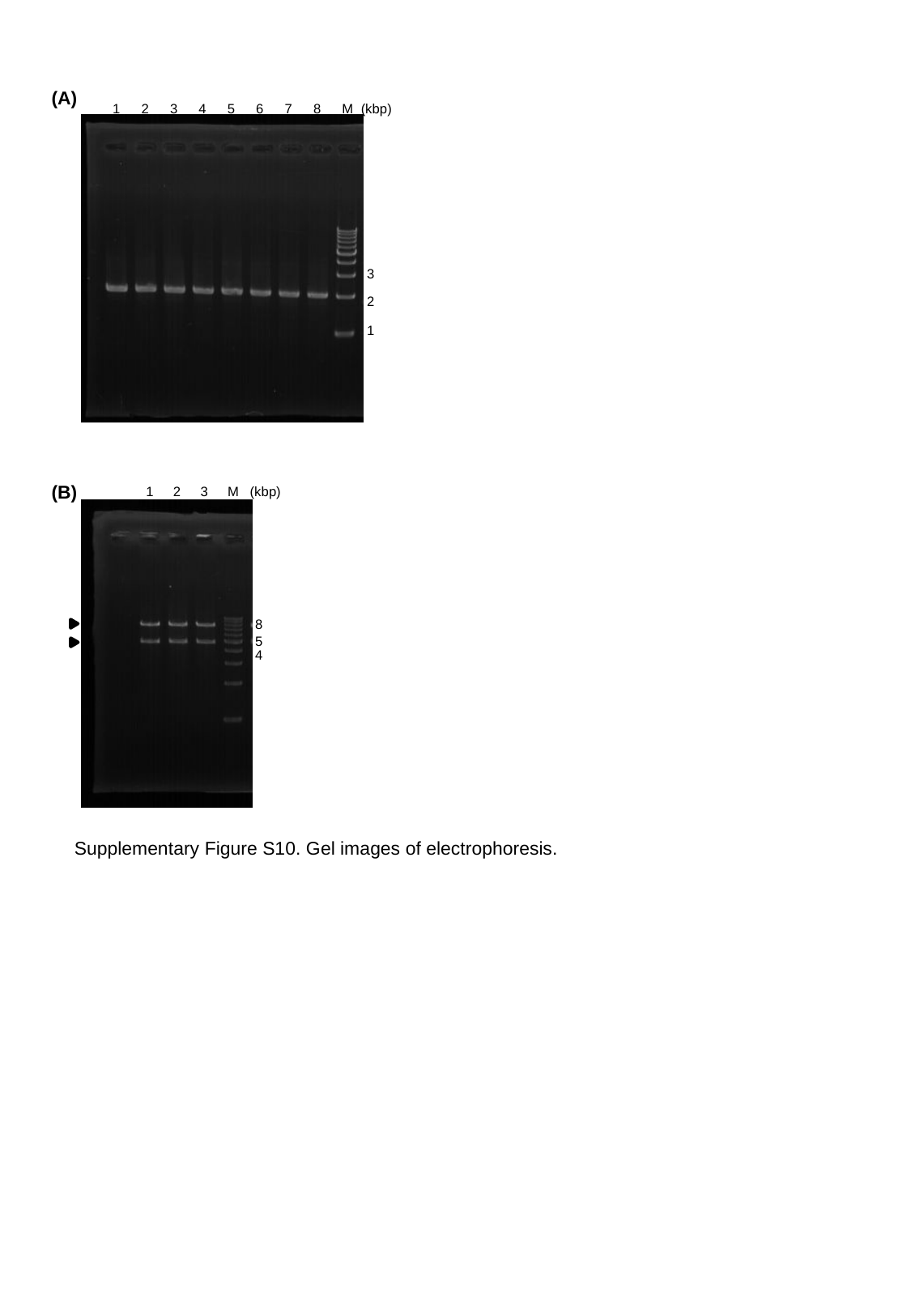

(A)
1
2
3
4
5
6
7
8
M
(kbp)
3
2
1
(B)
1
2
3
M
(kbp)
8
5
4
Supplementary Figure S10. Gel images of electrophoresis.

## Slide 11
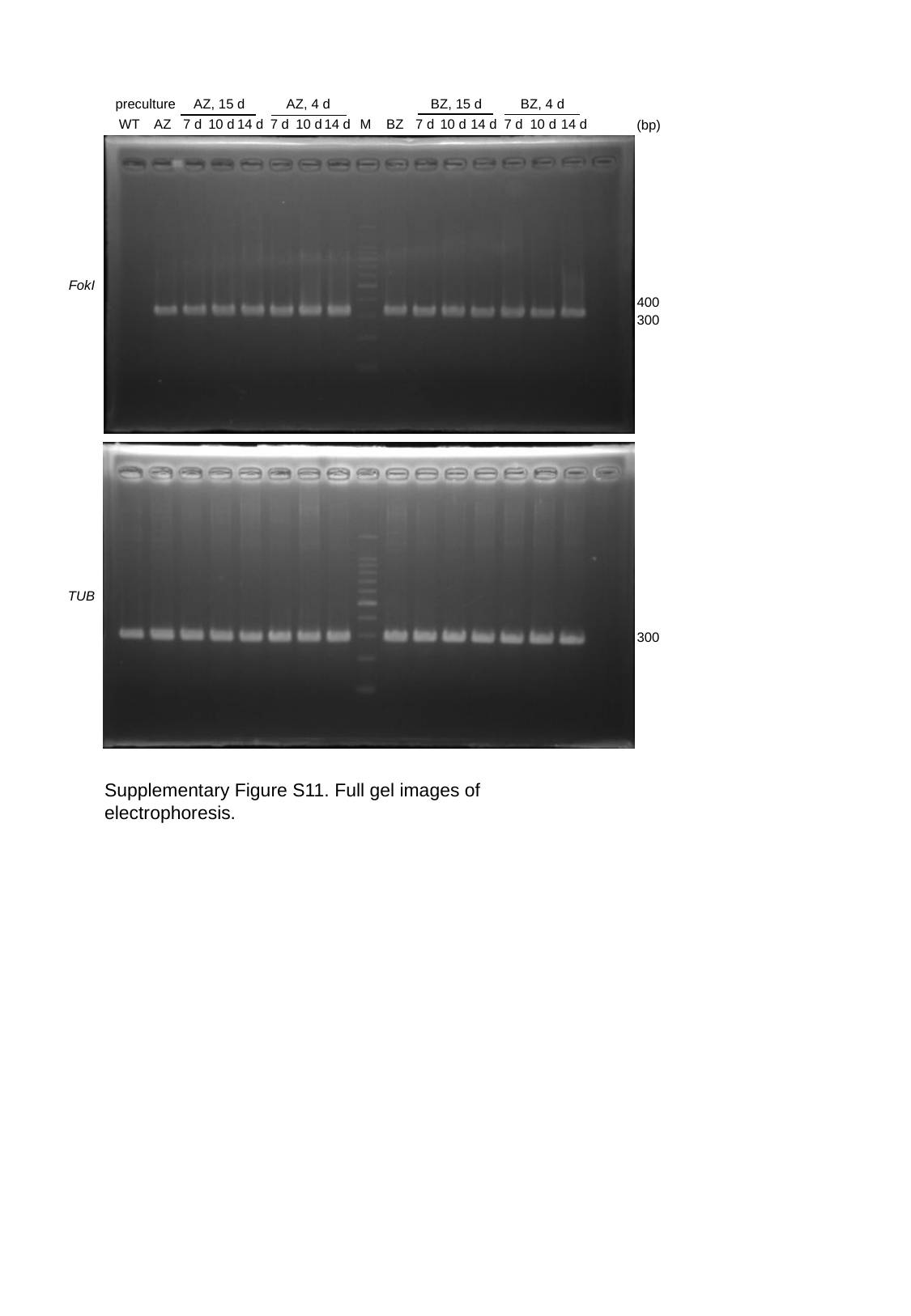

preculture
AZ, 15 d
AZ, 4 d
BZ, 15 d
BZ, 4 d
WT
AZ
7 d
10 d
14 d
7 d
10 d
14 d
M
BZ
7 d
10 d
14 d
7 d
10 d
14 d
(bp)
FokI
400
300
TUB
300
Supplementary Figure S11. Full gel images of electrophoresis.

## Slide 12
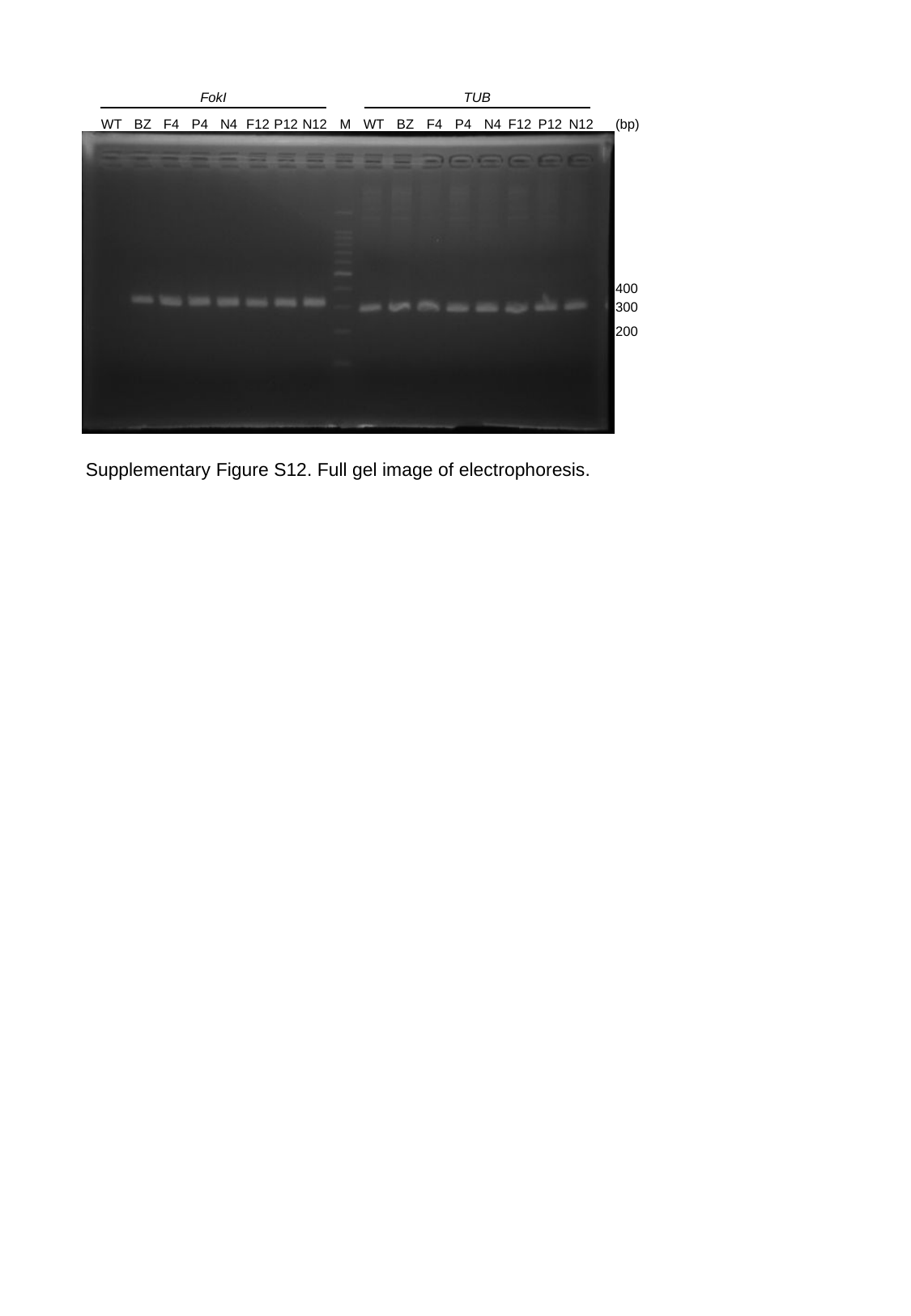

FokI
TUB
WT
BZ
F4
P4
N4
F12
P12
N12
M
WT
BZ
F4
P4
N4
F12
P12
N12
(bp)
400
300
200
Supplementary Figure S12. Full gel image of electrophoresis.
