# Supplementary material for: Genome editing with removable TALEN vectors harboring a yeast centromere and autonomous replication sequence in oleaginous microalga
Source: Sci Rep. 2022 Feb 15;12:2480. doi: 10.1038/s41598-022-06495-y (PMC8847555; doi:10.1038/s41598-022-06495-y)
Supplement: Supplementary file 3 — Supplementary Table S1. [file 41598_2022_6495_MOESM3_ESM.docx]

Supplementary Table S1 Primers used in this study.

| Primer | Sequence (5’-3’) |
| --- | --- |
| KanR-ins-F | TTCGGTTGTATTAATCGCCCAATCTGAATAATGTTACAACC |
| KanR-ins-R | ATTCGAGCTCGGTACGCAGCTCTGGCCCGTGTC |
| Am-delta-F | CTGTCAGACCAAGTTTACTCATA |
| Am-delta-R | AACTTGGTCTGACAGCCACCTGACGTCTAAGAAAC |
| KanR-clone-R | ATTAATACAACCGAAAAGAATAAGGAGAG |
| Marker-sequence-F | GTACCGAGCTCGAATTCACTG |
| KanR-dBsmBI-F | CCTGAGCAAGGCGAAATACGCGATCGCTGTTAAA |
| KanR-dBsmBI-R | CGCCTTGCTCAGGCGCAATCACG |
| ARS-ins-1-F | GTTCCTGGCCTTTTGAAGTGCCACCTGGGTCCTTTTC |
| ARS-ins-1-R | GGATAACGCAGGAAAGGATCGCTTGCCTGTAACTTACACG |
| ARS-cloning-1-F | TTTCCTGCGTTATCCCCTGATTCTG |
| ARS-cloning-1-R | CAAAAGGCCAGGAACCGTAAAAAGG |
| Ins-Marker-F | AATACAACCGAAAAGAATAAGGAGAGACA |
| Marker-cloning-R | CTTTTCGGTTGTATTGGTGTGAAATACCGCACAGATGCGT |
| FokI-F | CGTCAAGTCCGAACTCGAGGAGAAG |
| FokI-R | CTCCTCCACATATCGTTGCATCTCG |
| TALEN47-N-F | CGATTACAAGGACGACGACGACAAG |
| TALEN47-N-R | CCGGACCACTGTTTACCGACACC |
| KanR-F | GTGACGACTGAATCCGGTGAGAATG |
| KanR-R | TTTATGCCTCTTCCGACCATCAAGC |
| ZeoR-F | GACCACTCGGCGTACAGCTCGTC |
| ZeoR-R | ACGACGTGACCCTGTTCATCAGC |
| TUB-F | AGCATGGCATTGACTCCACC |
| TUB-R | AACGGCCTCGTTGTAGTACACG |
| KanR-sequence-F | CCTGATTGCCCGACATTATC |
| Marker-sequence-R | TGCCACCTGACGTCTAAGAA |
| NoNR-1-F | GGATCTTAGCGCGCAGTCGT |
| NoNR-1-R | GGTCACGGTTATGCGGTGGT |
