# Supplementary material for: Genome editing with removable TALEN vectors harboring a yeast centromere and autonomous replication sequence in oleaginous microalga
Source: Sci Rep. 2022 Feb 15;12:2480. doi: 10.1038/s41598-022-06495-y (PMC8847555; doi:10.1038/s41598-022-06495-y)
Supplement: Supplementary file 4 — Supplementary Table S2. [file 41598_2022_6495_MOESM4_ESM.docx]

Supplementary Table S2 Electroporation settings used with the Elepo21 electroporator for *Nannochloropsis*.

|  | Voltage (V) | Pulse length (mS) | Pulse interval  (mS) | Number of Pulse (Times) | Polarity |
| --- | --- | --- | --- | --- | --- |
| Poring Pulse | 500-2000 | 3.5 | 50 | 1 | + |
| Transfer Pulse | 100 | 50 | 50 | 3 | +/– |
