# Supplementary material for: Genome editing with removable TALEN vectors harboring a yeast centromere and autonomous replication sequence in oleaginous microalga
Source: Sci Rep. 2022 Feb 15;12:2480. doi: 10.1038/s41598-022-06495-y (PMC8847555; doi:10.1038/s41598-022-06495-y)
Supplement: Supplementary file 5 — Supplementary Table S3. [file 41598_2022_6495_MOESM5_ESM.docx]

Supplementary Table S3 Carrier DNA-free electroporation using an Elepo21 electroporator in *Nannochloropsis*.

|  | *Set values of Porting pulse | Measured values of resistance | Measured values of Poring pulse | | | Measured values of Transfer pulse | | | Efficiency of colony formation |
| --- | --- | --- | --- | --- | --- | --- | --- | --- | --- |
|  | Voltage (V) | (kΩ) | Voltage (V) | Current (A) | Total energy (J) | Voltage (V) | Current (A) | Total energy (J) | CFU  (colonies / μg DNAs) |
| 1 | Without electroporation, negative control | | | | | | | | 0 |
| 2 | 500 | 2.548 | 499.7 | 0.205 | 0.747 | 100.5 | 0.030 | 1.002 | 15 |
| 3 | 750 | 2.420 | 749.9 | 0.332 | 1.870 | 100.5 | 0.040 | 1.125 | 20 |
| 4 | 1,000 | 2.579 | 999.8 | 0.397 | 2.980 | 100.4 | 0.039 | 1.083 | 40 |
| 5 | 1,250 | 2.092 | 1,249.0 | 0.611 | 6.038 | 100.2 | 0.062 | 1.566 | 865 |
| 6 | 1,500 | 2.613 | 1,499.0 | 0.596 | 7.853 | 98.8 | 0.065 | 1.574 | 1010 |
| 7 | 1,750 | 2.384 | 1,698.0 | 0.753 | 12.990 | 98.8 | 0.117 | 2.415 | 1320 |
| 8 | 2,000 | 2.653 | Unmeasurable (occurring sparks) | | | | | | 20 |

*The other set values of Poring pulse and Transfer pulse are shown in Supplementary Table S2.
